# A graph-search framework for associating gene identifiers with documents

**DOI:** 10.1186/1471-2105-7-440

**Published:** 2006-10-10

**Authors:** William W Cohen, Einat Minkov

**Affiliations:** 1 Department of Machine Learning, Carnegie Mellon University, 5000 Forbes Avenue, Pittsburgh, PA 15213, USA; 2 Language Technology Institute, Carnegie Mellon University, 5000 Forbes Avenue, Pittsburgh, PA 15213, USA; 3 Center for Bioimage Informatics, Carnegie Mellon University, 5000 Forbes Avenue, Pittsburgh, PA 15213, USA

## Abstract

**Background:**

One step in the model organism database curation process is to find, for each article, the identifier of every gene discussed in the article. We consider a relaxation of this problem suitable for semi-automated systems, in which each article is associated with a ranked list of possible gene identifiers, and experimentally compare methods for solving this *geneId ranking *problem. In addition to baseline approaches based on combining named entity recognition (NER) systems with a "soft dictionary" of gene synonyms, we evaluate a graph-based method which combines the outputs of multiple NER systems, as well as other sources of information, and a learning method for reranking the output of the graph-based method.

**Results:**

We show that named entity recognition (NER) systems with similar F-measure performance can have significantly different performance when used with a soft dictionary for geneId-ranking. The graph-based approach can outperform any of its component NER systems, even without learning, and learning can further improve the performance of the graph-based ranking approach.

**Conclusion:**

The utility of a named entity recognition (NER) system for geneId-finding may not be accurately predicted by its entity-level F1 performance, the most common performance measure. GeneId-ranking systems are best implemented by combining several NER systems. With appropriate combination methods, usefully accurate geneId-ranking systems can be constructed based on easily-available resources, without resorting to problem-specific, engineered components.

## Background

### The GeneId curation task

Curators of biological databases, such as MGI [1] or Flybase [2], must read and analyze research papers in order to identify the experimental results that should be included in a database. As one example of this analysis process, Cohen and Hersh [3] describe the curation process for MGI as consisting of three stages. First, papers are selected according to whether or not they use mouse as a model organism. Second, the selected papers are *triaged *to determine if they contain curatable information. For MGI, papers must be curated when they contain evidence sufficient to assign a Gene Ontology term [4] to a specific gene. (These terms indicate some aspect of the molecular function, biological process, or cellular localization for a gene product.) Third, for articles that do contain such information, terms from Gene Ontology are assigned to specific genes, along with the appropriate evidence code.

To accomplish the final stage of curation, a curator must find, for each article being curated, the unique database identifier of every gene that will be annotated. Because the criteria for annotation is different for every model-organism database, it is useful to consider a slightly more general version of this task, namely *to find the unique database identifier of every gene mentioned in a document*. We will henceforth refer to this process as *geneId finding*.

GeneId finding is an important step in the curation process of many biological databases. It is also often a challenging task, because the nomenclature used for genes is inconsistent for many model organisms. In particular, authors often use novel variations of existing gene names in their papers; hence, even a large dictionary of genes and their synonyms will not contain all names appearing in a newly-published article. In addition, many existing gene names are ambiguous, corresponding to multiple unique gene identifiers.

GeneId finding is often broken down into two substeps: recognizing gene names in text, which is a a type of *named entity recognition *(NER); and then *normalization *of the recognized gene names, by mapping each name to the corresponding unique gene identifier. NER methods for genes have been widely investigated, and several public corpora for evaluation of NER techniques exist. Data for the task of geneId finding has become available for research more recently, in the form of the evaluation sets made available by the BioCreAtIvE challenge, task 1B [5,6].

### Automatic GeneId finding

Much prior work on biological text-mining is relevant to geneId finding. *Named entity recognition *(NER) is the task of identifying substrings in a document that correspond to particular entities. A large number of NER systems have been developed to identify genes in biomedical publications (e.g., [7-10]), utilizing a variety of techniques, including dictionary matching, rule-based approaches and machine learning methods. More specifically, most of these systems extract gene and protein names, without distinguishing between them, a task often called *gene-protein entity extraction*. NER systems have also been built that extract other entities from biological data, as well as relationships between entities (e.g. [9-13]).

NER systems are usually evaluated by their *F-measure performance*. F-measure is the geometric mean of *precision *(the fraction of all substrings extracted by the NER that are actually entity names) and *recall *(the fraction of all entity names that are extracted).

In some domains, the output of a NER system can be immediately used to solve the "id finding" problem for that domain, by simply looking up each extracted entity name in an appropriate database. For gene or protein entities, however, the "database lookup" step is non-trivial, for the reasons detailed earlier. Therefore, even a very accurate NER system for gene-protein entities may not be accurate at geneId finding; likewise, NER performance, as usually measured, is an imperfect proxy for performance on the geneId-finding task.

An important prior experimental study of geneId-finding was the BioCreAtIvE challenge, task 1B [5,6], which assembled common testbed problems and a common evaluation framework for this task. Three separate testbed problems were developed, one for each of three model organisms: yeast, mice, and fruitflies. In addition to sets of documents labeled with their associated geneId lists, the BioCreAtIvE challenge also assembled a *gene synonym list *for each organism – a list of all known gene identifiers, with several synonyms for each gene. (As the curation process defined here is limited to known gene identifiers, it may be incomplete in absolute terms.) Eight teams of researchers fielded geneId-finding systems and evaluated them on these common datasets.

Performance on the BioCreAtIvE datasets probably somewhat overstates performance in a realistic curation setting, as some simplifying assumptions were made in creating the challenge problem sets. Documents were abstracts, rather than full papers. It was assumed that a document was relevant to only one model organism, which was known to the geneId-finding system: thus, different versions of a system could be used for documents about different organisms, and no system needed to deal with the complications arising from a non-homogenous corpus, including documents relevant to multiple organisms, e.g., identically-named homologs. (This difficulty would not typically be relevant for a model-organism database, but could be relevant for other curation tasks, e.g., developing a BIND-like database of interactions.) Finally, the BioCreAtIvE task considers finding all genes *mentioned *in an abstract, not genes that should be included in a model organism database: in reality, only genes meeting certain (organism-specific) criteria are curated, and finding these particular genes is more important.

In spite of these simplifications, the BioCreAtIvE geneId-finding systems performed only moderately well for two of the three model organisms. (Many systems performed well for yeast, where the nomenclature for genes is fairly consistent.) For fly, the median and top F-measure of the systems fielded were 0.66 and 0.82, and for mouse, the median and top F-measure were 0.74 and 0.79, respectively.

### GeneId ranking

The results on the BioCreAtIvE challenge suggest to us that completely automatic systems are unlikely to be useful for geneId finding. A more realistic short-term goal would be a system that, given a document, provides a *ranked list of genes that might be discussed *by the document. Such a ranked list would be used as an aid for, rather than as a replacement of, a human curator. Henceforth we will call this sort of system a *geneId ranking *system. A good ranked list would have all or most of the correct gene identifiers near the top of the list, interspersed with only a few incorrect identifiers.

In this paper we experimentally compare a number of methods for geneId ranking. To evaluate such systems, we primarily use *mean average precision*, a measure adopted by the information retrieval community to evaluate the performance of search engines (which also return ranked lists of results). We consider first a baseline system in which gene-protein names extracted by a NER system are used to look up gene identifiers in a "soft dictionary" – i.e., a dictionary in which inexact matches between a dictionary entry and an extracted name are allowed. We show that NER systems with similar F-measures can have very different performance as part of such a geneId-ranking system. We then consider a scheme for geneId-ranking that *combines *the outputs of multiple NER systems – and potentially many other additional sources of evidence – in a natural way, by formulating geneId-ranking as a search process over graphs. In our formulation, the nodes of a graph consist of documents, terms (i.e., words from a document), gene identifiers, gene synonyms, extracted gene names, or lists of genes that co-occur in previously curated documents. We show that this graph-search formulation improves performance over any single NER-based system. Finally, we present a scheme for using machine learning to improve these results further. The learning approach can be used whenever there is training data in the form of documents paired with lists of correct gene identifiers.

## Results and discussion

### Datasets

To evaluate our approach, we use the data from the BioCreAtIvE challenge. Our primary focus is the mouse dataset, which proved to be hard for the BioCreAtIvE participants. This dataset consists of several parts.

• The *gene synonym list *consists of 183,142 synonyms for 52,594 genes.

• The *training data, development data*, and *test data *each consist of a collection of mouse-relevant MEDLINE abstracts, associated with the MGI geneIds for all of the genes that are mentioned in the abstract. These datasets contain 100, 50, and 250 abstracts, respectively.

• The *historical data *consists of 5000 mouse-relevant MEDLINE abstracts, each of which is associated with the MGI geneIds for all genes which are (a) associated with the article according to the MGI database, and (b) mentioned in the abstract, as determined by an automated procedure based on the gene synonym list. Notice that the list of geneIds associated with a historical-data abstract need *not *include all genes mentioned in the abstract; in fact, only 55% of the genes mentioned in these abstracts are found on this list [[6], Table 4].

**Table 4:** GeneId-Ranking Methods on Blind Test data

	MAP	Avg Max F1
*mouse test data*		

likely-prot + softTFIDF	0.368	0.421
possible-prot + softTFIDF	0.611	0.672
graph-based ranking	0.640	0.695
+ extra links & learning	0.711	0.755

Mean average precision and average maximal F1, for several geneId-ranking methods, on the 250 abstracts from the mouse test dataset.

For those familiar with BioCreAtIvE, our training set and development set are subsets of the BioCreAtIvE "devtest" set, and the historical data corresponds to the BioCreAtIvE "training" set. The terminology we use here more accurately reflects our use of the data. The test data is the same as the blind test set used in BioCreAtIvE. We did not manually examine the test set or use it for error analysis, program development, or debugging – its only purpose was as a final prospective test.

### NER systems

We used two closely related NER systems in our experiments. Both were trained using an off-the-shelf machine learning system for NER called Minorthird [14] on the YAPEX corpus [15], which contains 200 MEDLINE abstracts annotated for gene-protein entities.

To train the first NER system, we used Minorthird's default tokenizer and feature set, and Minorthird's implementation of a voted-perceptron based training scheme for HMMs due to Collins [16]. This simple algorithm has performed well on a number of previously-studied sequential learning tasks [16-18], including NER, and can be proven to converge under certain plausible assumptions [16].

This method learns a NER system with precision of 0.87 and recall of 0.62 on the YAPEX test set, which compares favorably with the performance of the original YAPEX system on the same data. We call this first system the *likely-protein extractor*, as it has fairly high precision and lower recall.

Precision and recall are very sensitive to slight errors in entity boundaries. An alternative pair of measures are *token precision *and *token recall*, defined as precision (respectively recall) of the decision associated with classifying a token as inside or outside an entity name. The likely-protein extractor has token precision of 0.95 and token recall of 0.65 on the YAPEX test set.

A second NER system was constructed by modifying the likely-protein extractor to improve its recall, at the expense of precision, by tuning it to optimize F_3 _(favoring recall – see Methods section for the complete *F*_*β *_formula) on the YAPEX test data [19]. This higher-recall protein extractor will be henceforth called the *possible-protein extractor*. The possible-protein extractor has field-level precision and recall of 0.47 and 0.83 on the YAPEX test set, and has token-level precision and recall of 0.49 and 0.97. (Note, however, that these are biased measurements since the test set was used in the optimization for *F*_3_, as described in the Methods section.)

**Figure 1:**
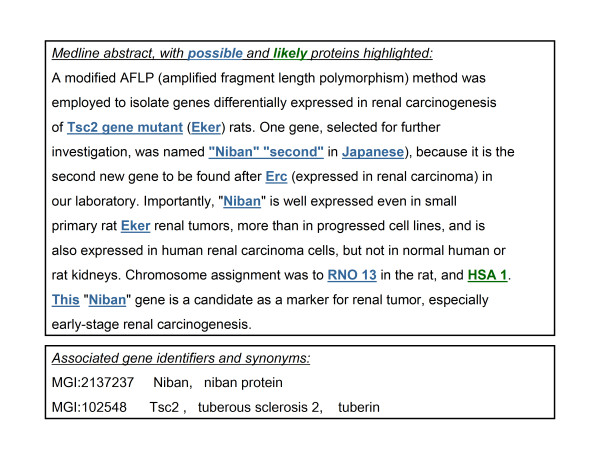
**Sample abstract**. A sample abstract from the mouse training dataset, together with the entries from the gene synonym list for the associated identifiers. Terms produced by the possible-protein extractor and likely-protein extractor (see text) are highlighted in the abstract. (The missing open parenthesis after the word "Niban" is a typographical error.)

Figure 1 gives an example of the output of these extractors on one of the development-set abstracts, and Table 1 summarizes the performance of these extractors on the mouse development data.

**Table 1:** Performance of NER Systems

	*Token-level*			*Field-level*		
	precis.	recall	F1	precis.	recall	F1
*development set*						

likely-protein	0.816	0.313	0.452	0.667	0.268	0.382
possible-protein	0.439	0.885	0.587	0.304	0.566	0.396
dictionary	0.501	0.469	0.484	0.245	0.439	0.314

*YAPEX test*						

likely-protein	0.949	0.648	0.770	0.872	0.621	0.725
possible-protein	0.490	0.974	0.652	0.472	0.825	0.600
YAPEX system				0.678	0.664	0.671

Performance of the NER systems on the mouse development corpus and the YAPEX test corpus. The row labeled "dictionary" is for simple dictionary lookup using the mouse synonym list. The row labeled "YAPEX system" is from [15].

Likely-protein and possible-protein still have high token precision and high token recall on the mouse data; they also have comparable F measures on this dataset. However, performance is much worse for both than on the YAPEX data; in fact, the F-measure performance of the two systems is only slightly better than merely looking for exact matches to an entry in the mouse synonym list (recorded as "dictionary" in the table).

Presumably most of the performance degradation is due to systematic differences between the data on which the NER system was tested and the data on which it was trained; similar degradation has been documented elsewhere in transferring learned gene-protein NER systems from one distribution of documents to another [20]. Unfortunately, no large dataset of abstracts from the same distribution as the mouse development data seems to exist. (The BioCreAtIvE workshop distributed a subset of the GENETAG corpus for Task 1A. GENETAG contains a sample of MEDLINE sentences enriched for gene-protein entity names, but not specific to the mouse model organism). In the remainder of this paper, we will describe how even such low-performance NER systems can be used to generate accurate geneId-ranking systems.

### GeneId ranking by soft matching

The row labeled "dictionary" in Table 1, as well as the experiences of several BioCreAtIvE participants, shows that requiring exact matches to the synonym list will lead to recall problems. We thus incorporated certain approximate string matching techniques in our geneId-ranking systems.

Specifically, we used a similarity metric for strings called *softTFIDF*. In prior work [21], SoftTFIDF was shown to be, on average across several different string-matching problems, the most effective of several different heuristic similarity metrics. SoftTFIDF breaks strings into tokens which are then weighted, using a statistical weighting scheme widely used in information retrieval called TFIDF. The similarity of *s *and *t *is determined by the weight of the tokens *w *contained in *s *that are are highly similar to some token *w' *in *t *(i.e., the order of tokens is ignored by this measure). A second similarity measure called Jaro-Winkler is used to measure token similarity; Jaro-Winkler is an edit-distance-like measure used widely for record-linkage tasks involving personal names. We note that the mouse dataset has many multi-token gene names: the average length of a gene name in the synonym list is 2.77 words, and 19% of the gene names are five words long or longer [6].

A simple baseline approach to implementing geneId finding, given a NER system, is to soft-match extracted gene names against the synonym list. Specifically, given a document *d*, one takes each candidate gene name *s *extracted from *d *by a NER system, finds the synonym *s' *in the gene synonym list that is most similar to *s*, and adds the gene associated with *s' *to the proposed gene list for *d*. Gene identifiers on the list are ranked according to the number of extracted names that were mapped to them.

We implemented and evaluated this approach for the likely-protein and possible-protein extractors. The performance of these systems on the mouse development data is shown in Table 2.

**Table 2:** GeneId-Ranking Methods on Development Data

	Mean Average Precision (MAP)
*mouse development data*	

likely-prot + softTFIDF	0.450
possible-prot + softTFIDF	0.626

graph-based ranking	0.513
+ extra links	0.730
+ extra links & learning	0.807

Mean average precision of several geneId-ranking methods on the 50 abstracts from the mouse development dataset. The first two lines are NER systems, coupled with using softTFIDF to matching extracted names with gene synonyms. The graph-based ranking method (see text) ranks geneIds by proximity to a query abstract in a graph that includes the results of both NER systems.

Here and elsewhere, we evaluate the ranked lists by *mean average precision*. To motivate this measurement, consider a ranked list that has *n *correct entries at ranks *k*_1_, ..., *k*_*n*_, and assume that the end user will scan down the list of answers and stop at some particular "target answer" *k*_*i *_that she finds to be of interest. One would like the density of correct answers up to rank *k*_*i *_to be high: to formalize this, define the *precision at rank k, prec*(*k*), to be the number of correct entries up to rank *k*, divided by *k *– i.e., the precision of the list up to rank *k*. The *non-interpolated average precision *of the ranking is simply the average of *prec*(*k*) for each position *k*_*i *_that holds a correct entry:

Average Precision=1n∑i=1nprec(ki)
 MathType@MTEF@5@5@+=feaafiart1ev1aaatCvAUfKttLearuWrP9MDH5MBPbIqV92AaeXatLxBI9gBaebbnrfifHhDYfgasaacH8akY=wiFfYdH8Gipec8Eeeu0xXdbba9frFj0=OqFfea0dXdd9vqai=hGuQ8kuc9pgc9s8qqaq=dirpe0xb9q8qiLsFr0=vr0=vr0dc8meaabaqaciaacaGaaeqabaqabeGadaaakeaacqWGbbqqcqWG2bGDcqWGLbqzcqWGYbGCcqWGHbqycqWGNbWzcqWGLbqzcqqGGaaiieGacqWFqbaucqWFYbGCcqWGLbqzcqWGJbWycqWGPbqAcqWGZbWCcqWGPbqAcqWGVbWBcqWGUbGBcqGH9aqpdaWcaaqaaiabigdaXaqaaiabd6gaUbaadaaeWbqaaiabdchaWjabdkhaYjabdwgaLjabdogaJjabcIcaOiabdUgaRnaaBaaaleaacqWGPbqAaeqaaOGaeiykaKcaleaacqWGPbqAcqGH9aqpcqaIXaqmaeaacqWGUbGBa0GaeyyeIuoaaaa@5751@

As an example, consider a ranked list of items (gene identifiers), where the items at ranks 1,2,5 are correct and those at ranks 3,4 are not. The precision at ranks 1 and 2 equals 1.0, and the precision at the next correct item is 0.6 (since there are 3 correct answers before rank 5). The non-interpolated average precision on this ranked list is thus (1 + 1 + 0.6)/3 = 0.87.

Elsewhere, it has been noted that *prec*(*k*_*i*_)can be viewed as a ratio *m*_*i*,*actual*_/*m*_*i*,*opt*_, where *m*_*i*,*opt *_is the number of entries the user must examine to find the *i*-th correct entry in an optimal ranked list, and *m*_*i*,*actual *_is the analogous number for the actual ranked list [22]. Thus, non-interpolated average precision can also be interpreted as a measure of the additional work imposed on the user by a suboptimal ranking – e.g., an average precision of 0.5 means that the user must examine twice as many list entries as needed, on average.

In our ranking systems, it may happen that some correct answers do not appear in the ranking at all: in this case, we follow standard practice and define *prec*(*k*_*i*_) for that answer to be zero. (Returning to our example, if there were a fourth answer that did not appear anywhere on the list, then the average precision would be (1 + 1 + 0.6 + 0)/4 = 0.65.) If there are no correct answers for a problem, we define the average precision of any ranking to be 1.0. Finally, *mean average precision *(MAP) averages non-interpolated average precision across a number of problems. In Table 2, the MAP scores are averaged across the 50 abstracts in the mouse development set.

For this dataset, the geneId ranker based on possible-protein performs statistically significantly better (with *z *= 3.1, *p *< 0.005, using a two-tailed paired test on the individual non-interpolated average precision scores of the 50 problems) than the one based on likely-protein: the ranker using the possible-protein extractor yields a MAP score of 0.63, compared to a MAP score of 0.45 using likely-protein (Table 2). This result might be viewed as surprising, as the F1 scores of the likely-protein extractor are comparable to, or better, than those of the possible-protein extractor: on the YAPEX test set, the two methods have F1 scores of 0.73 and 0.60, respectively; and on the development data, the two methods have F1 of 0.38 and 0.40, respectively (Table 1).

We thus observe that for this dataset, the widely-used F1 score does not predict the relative performance of these two NER systems, when they are used as components of a geneId-ranking using soft matching. This may be because in a geneId finding pipeline, NER systems that have higher recall are preferable, as the subsequent process of normalization to unique ids may downweight gene names that are incorrect. More generally, F1 score may be an imperfect measure of performance for any NER system that is used in the context of a larger problem – an observation which has implications on the proper evaluation of such systems.

### Graph search for GeneId ranking

#### Motivation

**Figure 2:**
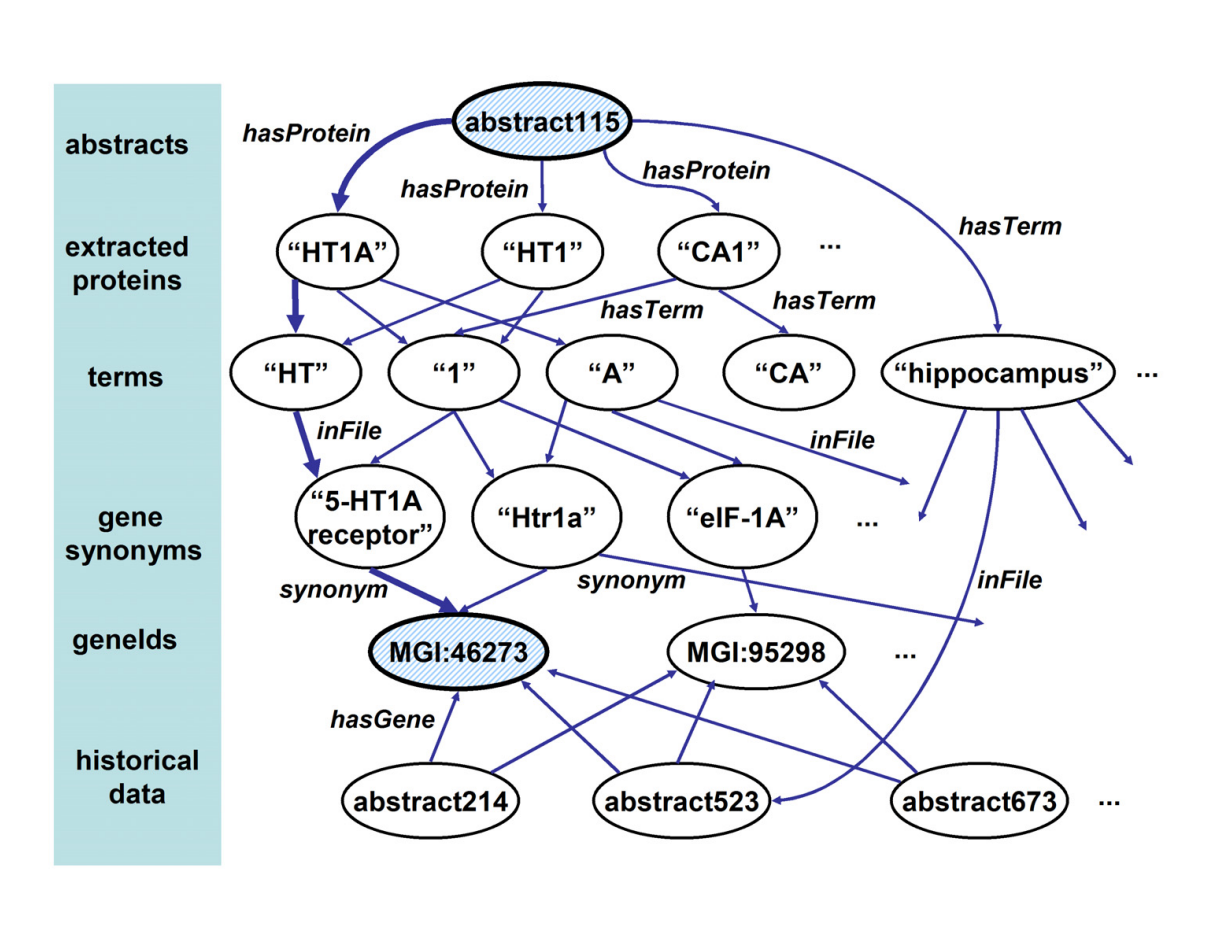
**Graph used for geneId-ranking**. Part of a simplified version of the graph used for geneId ranking. In the ranking system, geneIds are ranked by a certain notion of proximity in the graph (see text). The graph is simplified by not showing inverse links, and by showing only a single NER-related link type.

We will now describe a scheme for combining multiple NER systems into a single unified geneId-ranking system. The core of the idea is to first represent all information as a labeled directed graph which includes the test abstracts, the extracted names, the synonym list, and the historical data; and then to use *proximity in the graph *for ranking. A simplified version of the graph we used is illustrated in Figure 2. Nodes in this graph can be either *files, strings *or *terms*; alternatively, nodes can belong to a *user-defined type*. Abstracts and gene synonyms are represented as *file *and *string *nodes, respectively. Files are linked to the *terms *(i.e., the words) that they contain by edges labeled *hasTerm*, and terms are linked to the files that contain them by edges labeled *inFile*. *String *nodes are linked with terms (i.e., words) in the same way (conceptually, they are simply short files). File nodes are also linked to *string *nodes corresponding to the output of a NER system on that file: here, an abstract is linked to strings extracted by the likely-protein extractor by edges labeled *hasLikelyProtein*, and linked to strings extracted by the possible-protein extractor by edges labeled *hasPossibleProtein*. (For simplicity, in Figure 2 we show only one such edge type, which is labeled *hasProtein*.)

The nodes and edges listed above are all produced automatically by our system as it reads in NER-annotated files. (The graph structure produced is slightly more complex than suggested by Figure 2, as noted in the Methods section.) In our experiments we annotate and store all the historical-data abstracts, as well as the development- or test-data abstracts. The graph also contains one user-defined node type, and several user-defined edges: nodes of the type *geneIds *are created from the gene synonym list, and for every synonym *s *associated with the *geneId x*, we create a *string *node *y*_*s *_that is linked to the node for *x *via an edge labeled *synonym*. For each historical-data abstract, we also have a list of gene identifiers that are known to co-occur in that abstract; thus we link historical-data abstract nodes to the appropriate *geneId *nodes with an edge labeled *hasGene*. (Hence, we are using the historical-data gene lists as background data, which may guide the geneId ranking process, but not as training data for the learner. Recall that the lists of geneIds associated with historical data are incomplete.) Although edges in the graph are directed, all of the edge labels described above also have inverses: for instance, there is an edge labeled *synonym*^-1 ^from a synonym node to the geneId(s) to which it corresponds.

Given this graph, gene identifiers for an abstract are generated by traversing the graph away from the abstract, and looking for *geneId *nodes that are "close" to the abstract according to the proximity measure that we will formally describe below. Intuitively, this measure measures the similarity of two nodes by the weighted sum of all paths that connect the nodes. Shorter paths will be weighted higher than longer paths. Also, paths that use more frequent edge-labels (like *hasPossibleProtein*) are weighted lower than paths with less frequent edge-labels (like *hasLikelyProtein*). More specifically, an edge in a path from *x *to *y *labeled ℓ will have lower weight if there are many other edges from from *x *labeled ℓ.

As an example, consider the two shaded nodes in Figure 2, and the shaded path from *abstract115 *hasLikelyProtein→
 MathType@MTEF@5@5@+=feaafiart1ev1aaatCvAUfKttLearuWrP9MDH5MBPbIqV92AaeXatLxBI9gBaebbnrfifHhDYfgasaacH8akY=wiFfYdH8Gipec8Eeeu0xXdbba9frFj0=OqFfea0dXdd9vqai=hGuQ8kuc9pgc9s8qqaq=dirpe0xb9q8qiLsFr0=vr0=vr0dc8meaabaqaciaacaGaaeqabaqabeGadaaakeaadaWfqaqaaiabdIgaOjabdggaHjabdohaZjabdYeamjabdMgaPjabdUgaRjabdwgaLjabdYgaSjabdMha5Hqaciab=bfaqjab=jhaYjabd+gaVjabdsha0jabdwgaLjabdMgaPjabd6gaUbWcbaWaa4akaWqabeaaaSGaayPKHaaabeaaaaa@4424@*"HTA1" *hasTerm→
 MathType@MTEF@5@5@+=feaafiart1ev1aaatCvAUfKttLearuWrP9MDH5MBPbIqV92AaeXatLxBI9gBaebbnrfifHhDYfgasaacH8akY=wiFfYdH8Gipec8Eeeu0xXdbba9frFj0=OqFfea0dXdd9vqai=hGuQ8kuc9pgc9s8qqaq=dirpe0xb9q8qiLsFr0=vr0=vr0dc8meaabaqaciaacaGaaeqabaqabeGadaaakeaadaWfqaqaaiabdIgaOjabdggaHjabdohaZjabdsfaujabdwgaLjabdkhaYjabd2gaTbWcbaWaa4akaWqabeaaaSGaayPKHaaabeaaaaa@37EA@*"HT" *inFile→
 MathType@MTEF@5@5@+=feaafiart1ev1aaatCvAUfKttLearuWrP9MDH5MBPbIqV92AaeXatLxBI9gBaebbnrfifHhDYfgasaacH8akY=wiFfYdH8Gipec8Eeeu0xXdbba9frFj0=OqFfea0dXdd9vqai=hGuQ8kuc9pgc9s8qqaq=dirpe0xb9q8qiLsFr0=vr0=vr0dc8meaabaqaciaacaGaaeqabaqabeGadaaakeaadaWfqaqaaiabdMgaPjabd6gaUjabdAeagjabdMgaPjabdYgaSjabdwgaLbWcbaWaa4akaWqabeaaaSGaayPKHaaabeaaaaa@3667@*"5-HT1A receptor" *synonym→
 MathType@MTEF@5@5@+=feaafiart1ev1aaatCvAUfKttLearuWrP9MDH5MBPbIqV92AaeXatLxBI9gBaebbnrfifHhDYfgasaacH8akY=wiFfYdH8Gipec8Eeeu0xXdbba9frFj0=OqFfea0dXdd9vqai=hGuQ8kuc9pgc9s8qqaq=dirpe0xb9q8qiLsFr0=vr0=vr0dc8meaabaqaciaacaGaaeqabaqabeGadaaakeaadaWfqaqaaiabdohaZjabdMha5jabd6gaUjabd+gaVjabd6gaUjabdMha5jabd2gaTbWcbaWaa4akaWqabeaaaSGaayPKHaaabeaaaaa@387C@*MGI:46273*. This path would probably have greater weight than a corresponding path in which the link labeled *hasLikelyProtein *was replaced with one labeled *hasPossibleProtein*, because *abstract115 *will be linked to more "possible" proteins than "likely" proteins. Likewise, replacing the term "HT" with the term "A" in the path would greatly reduce its weight, as there are many more edges of the form *"A" *inFile→
 MathType@MTEF@5@5@+=feaafiart1ev1aaatCvAUfKttLearuWrP9MDH5MBPbIqV92AaeXatLxBI9gBaebbnrfifHhDYfgasaacH8akY=wiFfYdH8Gipec8Eeeu0xXdbba9frFj0=OqFfea0dXdd9vqai=hGuQ8kuc9pgc9s8qqaq=dirpe0xb9q8qiLsFr0=vr0=vr0dc8meaabaqaciaacaGaaeqabaqabeGadaaakeaadaWfqaqaaiabdMgaPjabd6gaUjabdAeagjabdMgaPjabdYgaSjabdwgaLbWcbaWaa4akaWqabeaaaSGaayPKHaaabeaaaaa@3667@*y *in the graph than edges of the form *"HT" *inFile→
 MathType@MTEF@5@5@+=feaafiart1ev1aaatCvAUfKttLearuWrP9MDH5MBPbIqV92AaeXatLxBI9gBaebbnrfifHhDYfgasaacH8akY=wiFfYdH8Gipec8Eeeu0xXdbba9frFj0=OqFfea0dXdd9vqai=hGuQ8kuc9pgc9s8qqaq=dirpe0xb9q8qiLsFr0=vr0=vr0dc8meaabaqaciaacaGaaeqabaqabeGadaaakeaadaWfqaqaaiabdMgaPjabd6gaUjabdAeagjabdMgaPjabdYgaSjabdwgaLbWcbaWaa4akaWqabeaaaSGaayPKHaaabeaaaaa@3667@*y*.

Paths through the historical data also contribute to the final ranking. For instance, the proximity of *abstract115 *and *MGI:46273 *is reinforced by the fact that the term "hippocampus" occurs in *abstract115 *and also in the historical-data abstract *abstract523*, which in turn contains the geneId *MGI:46273*. This evidence would be stronger if "hippocampus" were a less frequent term, and weaker if it were a more frequent term. Notice that the graph also assigns some non-zero similarity to geneId nodes that are not similar to any extracted string, but simply contain a term in *abstract115*. It also assigns non-zero similarity to geneIds that co-occur in the historical-data abstracts with genes extracted by the NER systems.

More formally, similarity between two nodes is defined by a *lazy walk process*. To walk away from a node *x*, one first picks an edge label ℓ; then, given ℓ, one picks a node *y *such that x→ℓy
 MathType@MTEF@5@5@+=feaafiart1ev1aaatCvAUfKttLearuWrP9MDH5MBPbIqV92AaeXatLxBI9gBaebbnrfifHhDYfgasaacH8akY=wiFfYdH8Gipec8Eeeu0xXdbba9frFj0=OqFfea0dXdd9vqai=hGuQ8kuc9pgc9s8qqaq=dirpe0xb9q8qiLsFr0=vr0=vr0dc8meaabaqaciaacaGaaeqabaqabeGadaaakeaacqWG4baEdaGdKaWcbaGaeS4eHWgabeGccaGLsgcacqWG5bqEaaa@3269@. We assume that the probability of picking the label ℓ is uniform over all label types *L*(*x*) that label edges leaving *x*. For most node and edge types, after ℓ is picked, *y *is chosen uniformly from the set of all *y *such that x→ℓy
 MathType@MTEF@5@5@+=feaafiart1ev1aaatCvAUfKttLearuWrP9MDH5MBPbIqV92AaeXatLxBI9gBaebbnrfifHhDYfgasaacH8akY=wiFfYdH8Gipec8Eeeu0xXdbba9frFj0=OqFfea0dXdd9vqai=hGuQ8kuc9pgc9s8qqaq=dirpe0xb9q8qiLsFr0=vr0=vr0dc8meaabaqaciaacaGaaeqabaqabeGadaaakeaacqWG4baEdaGdKaWcbaGaeS4eHWgabeGccaGLsgcacqWG5bqEaaa@3269@. (Two exceptions to this default scheme are edges of type ℓ = *hasTerm *and ℓ = *inFile*. As detailed in the Methods section, we use scores from an open-source full-text retrieval engine to weight these edge types.) At each step in a lazy graph walk, there is also some probability γ of simply stopping.

Conceptually, the edge weights above define the probability of moving from a node *x *to some other node *z*, which we will denote as *Q*(*z*|*x*). In our graph-based geneId-ranking system, *x *is an abstract for which gene identifiers are to be found. We approximate *Q*(*z*|*x*) for some set of nodes *z*, and then filter out those nodes *z' *that have type *geneId*, and order them by *Q*(*z'*|*x*).

The similarity metric *Q*(*z*|*x*) is a generalization of the *heat diffusion kernels on graphs *of Kondor and Lafferty [23]. These have in turn been shown to generalize Gaussian kernels, in the sense that the continuous limit of heat-diffusion kernels on a two-dimensional grid is a Gaussian kernel.

We note that this graph framework is general, and can be used for various other types of queries. For example, starting the random walk from a particular extracted protein name *x *can be applied to retrieve a ranked list of related geneIds that is specific to that protein; alternatively, one could start the random walk with a probability distribution which includes both an extracted protein *x *and the abstract *y *in which it appears, thus modeling the context of *x*. In another work [24] it has been shown that in the latter scenario the graph walk can be effective in assigning identifiers to specific personal names appearing in documents.

#### Engineering the graph for better performance

As shown in Table 2, the graph-based approach has performance intermediate between the two baseline systems. However, the baseline approaches include some information which is not available in the graph. For instance, the baseline systems can compute the soft TFIDF similarity of extracted protein names and gene synonyms. This information can be made available in the graph by inserting extra edges labeled *proteinToSynonym *between each extracted protein string *x *and all synonyms *y *that would be compared to *x *using the inverted-index based soft TFIDF matching scheme described in the Methods section. We used a non-uniform weighting scheme for these links, weighting edges according to the similarity of *y to x *under soft TFIDF.

Each baseline method also algorithmically incorporates the knowledge that a group of paths through the graph are important – namely, paths from an abstract to a NER-extracted string to a synonym (via a *proteinToSynonym *edge) to a *geneId *node. To make this sort of knowledge available in the graph, one can insert "short-cut" edges in the graph that directly link abstracts *x *to *geneId *nodes *y *that are the endpoint of these paths. We inserted two varieties of these "short-cut" edges. For the first variety, we inserted edges to connect abstracts to a single *y *for each extracted string (as is done by the baseline extractors). For the second, we inserted edges to connect abstracts to all nodes *y *that are reachable via a NER step followed by a *proteinToSynonym *link and then a *synonym *link, and weighted the nodes *y *reachable by these edges proportionally to the weight of the associated *proteinToSynonym *link.

As Table 2 shows, graph search with the augmented graph does indeed improve MAP performance on the mouse development data: the MAP score of 0.73 is better than the original graph, and also better than either of the baseline methods described above. One reason for the improved performance over the NER systems is that the graph also includes additional information in the form of labeled historical abstracts. An important advantage of the graph-search framework is that it can integrate this and other types of evidence.

### Learning to rank

We next consider the topic of improving the results of Table 2, by tuning the graph-based ranking using learning techniques. There has been substantial previous research in learning to rank objects – indeed, the learning method used for NER is based on one such method. The immediate obstacle to applying such techniques to this problem is determining how to describe a ranked item *z *with a feature vector. In general, designing these features would require a domain-specific human engineering effort. Here we will describe a scheme for constructing feature vectors automatically using the graph.

Intuitively, it would be desirable for this feature vector to summarize the lazy walk process which led to the ranking. We assume a vector **f **of primitive feature functions that describe the individual edges in a graph. For instance, one such feature function might be

f17(x→ℓy)={1if T(x)=string and ℓ−protToSyn0else
 MathType@MTEF@5@5@+=feaafiart1ev1aaatCvAUfKttLearuWrP9MDH5MBPbIqV92AaeXatLxBI9gBaebbnrfifHhDYfgasaacH8akY=wiFfYdH8Gipec8Eeeu0xXdbba9frFj0=OqFfea0dXdd9vqai=hGuQ8kuc9pgc9s8qqaq=dirpe0xb9q8qiLsFr0=vr0=vr0dc8meaabaqaciaacaGaaeqabaqabeGadaaakeaacqWGMbGzdaahaaWcbeqaaiabigdaXiabiEda3aaakiabcIcaOiabdIha4naaoqcaleaacqWItecBaeqakiaawkziaiabdMha5jabcMcaPiabg2da9maaceqabaqbaeaabiGaaaqaaiabigdaXaqaaiabbMgaPjabbAgaMjabbccaGiabdsfaujabcIcaOiabdIha4jabcMcaPiabg2da9iabdohaZjabdsha0jabdkhaYjabdMgaPjabd6gaUjabdEgaNjabbccaGiabbggaHjabb6gaUjabbsgaKjabbccaGiabloriSjabgkHiTiabdchaWjabdkhaYjabd+gaVjabdsha0jabdsfaujabd+gaVjabdofatjabdMha5jabd6gaUbqaaiabicdaWaqaaiabbwgaLjabbYgaSjabbohaZjabbwgaLbaaaiaawUhaaaaa@6644@

In our experiments, we constructed one such feature function for each possible combination of a source node type *T*(*x*)and edge label ℓ.

We then recursively define another vector function **F **which aggregates these primitive feature functions by computing the expected value of the primitive features appearing in any edge of the graph (see the Methods section for details). This vector **F**(*z*|*x*) summarizes and aggregates the features associated with every edge involved in the walk from *x *to *z*. Thus, it is a useful description of an element *z *obtained as the result of a query *x *(e.g., a *geneId *node *z *obtained as the result of submitting a document *x *to a geneId-ranking system).

Once feature vectors are constructed, any of several existing methods for learning-to-rank can be employed. The learning algorithm that we use is based on *voted perceptrons *[25,16]. We begin with weight vector **W**_0 _= **0**. We then iterate through each abstract *x *in a training set, and use the graph-based geneId-ranking system to produce candidate *geneId *nodes *z*_1_,..., *z*_*m*_. We look in this ranking for a node zj−
 MathType@MTEF@5@5@+=feaafiart1ev1aaatCvAUfKttLearuWrP9MDH5MBPbIqV92AaeXatLxBI9gBaebbnrfifHhDYfgasaacH8akY=wiFfYdH8Gipec8Eeeu0xXdbba9frFj0=OqFfea0dXdd9vqai=hGuQ8kuc9pgc9s8qqaq=dirpe0xb9q8qiLsFr0=vr0=vr0dc8meaabaqaciaacaGaaeqabaqabeGadaaakeaacqWG6bGEdaqhaaWcbaGaemOAaOgabaGaeyOeI0caaaaa@30A0@ that is incorrect (for *x*) but that is ranked above some correct node zk+
 MathType@MTEF@5@5@+=feaafiart1ev1aaatCvAUfKttLearuWrP9MDH5MBPbIqV92AaeXatLxBI9gBaebbnrfifHhDYfgasaacH8akY=wiFfYdH8Gipec8Eeeu0xXdbba9frFj0=OqFfea0dXdd9vqai=hGuQ8kuc9pgc9s8qqaq=dirpe0xb9q8qiLsFr0=vr0=vr0dc8meaabaqaciaacaGaaeqabaqabeGadaaakeaacqWG6bGEdaqhaaWcbaGaem4AaSgabaGaey4kaScaaaaa@3097@. For every such node pair, we set

**W**_*t *_= **W**_*t *_+ **F**(zk+
 MathType@MTEF@5@5@+=feaafiart1ev1aaatCvAUfKttLearuWrP9MDH5MBPbIqV92AaeXatLxBI9gBaebbnrfifHhDYfgasaacH8akY=wiFfYdH8Gipec8Eeeu0xXdbba9frFj0=OqFfea0dXdd9vqai=hGuQ8kuc9pgc9s8qqaq=dirpe0xb9q8qiLsFr0=vr0=vr0dc8meaabaqaciaacaGaaeqabaqabeGadaaakeaacqWG6bGEdaqhaaWcbaGaem4AaSgabaGaey4kaScaaaaa@3097@|*x*) - **F**(zj−
 MathType@MTEF@5@5@+=feaafiart1ev1aaatCvAUfKttLearuWrP9MDH5MBPbIqV92AaeXatLxBI9gBaebbnrfifHhDYfgasaacH8akY=wiFfYdH8Gipec8Eeeu0xXdbba9frFj0=OqFfea0dXdd9vqai=hGuQ8kuc9pgc9s8qqaq=dirpe0xb9q8qiLsFr0=vr0=vr0dc8meaabaqaciaacaGaaeqabaqabeGadaaakeaacqWG6bGEdaqhaaWcbaGaemOAaOgabaGaeyOeI0caaaaa@30A0@|*x*)

After learning, the weight vector **W **can be used to re-rank the output of the graph-based ranker as follows. Given a node *x*, the original graph-based geneId ranker is used to produce candidate nodes *z*_1_, ..., *z*_*m*_. These nodes are then re-ordered by decreasing values of **W**·**F**(*z*_*j*_|*x*).

As Table 2 shows, the learning approach does improve performance on the mouse development data. In combination, the techniques described have improved MAP performance to 0.807. This represents an improvement of nearly 80% over the baseline method of using the "best" NER method (according to F1 measure) with a soft dictionary.

## Results

As a final prospective test, we applied the two baseline methods to the 250-abstract mouse test data. We compared their performance to the graph-based search method combined with a reranking postpass learned from the 100-abstract mouse training data. The performance of these methods is summarized in Table 4.

All of the methods perform somewhat less well than on the 50-abstract mouse development set. This is probably due to variation in the two samples – for instance, the test-set abstracts contain somewhat more proteins on average (2.2 proteins/abstract) than the development-set abstracts (1.7 proteins/abstract).

**Figure 3:**
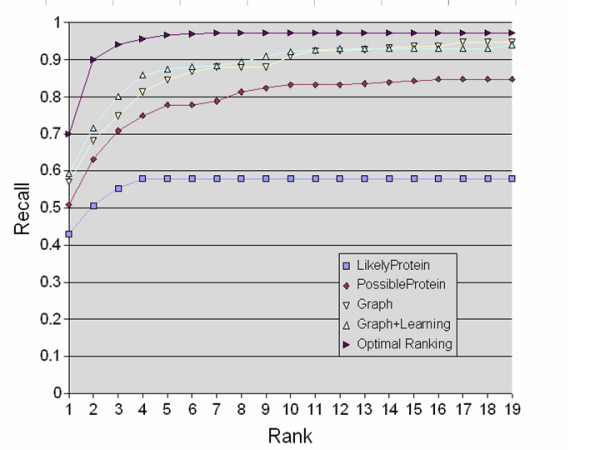
**Average recall vs rank for geneId-ranking**. Average recall for lists truncated at rank *r *as a function of *r*. The lines labeled *LikelyProtein *and *PossibleProtein *are for the baseline methods. The line labeled *optimal ranking *is for an ideal re-ranking of the graph-based search's output – one that places all correct protein identifiers first in the ranking.

Figure 3 gives another overview of performance, by showing recall as a function of rank for the mouse development data. For example, using the *likely protein *extractor with soft TFIDF matching, about 50% of the correct proteins will be found in the first two elements of the list; using the *possible protein *extractor the same way, about 63% of correct proteins will be found in the top two elements. Using a learned ranker to re-order the top 100 elements returned by the graph walk, 68% of correct proteins will be found in the top two elements of the list, 84% will be found in the top five elements, and more than 90% will be found in the top ten elements.

As shown in the graph, an ideal re-ranking would find 70% of the correct proteins by rank one, rather than rank two, and could achieve almost 97% recall by rank five, rather than only 84%. The graph walk results are encouraging, compared to this optimal curve. In particular, the graph walk reaches nearly maximal recall within the top 15–20 ranks.

This performance might be further improved by better learning methods or (more readily) by extending the graph to include more information sources. (It should be recalled that this result was obtained requiring as resources only a synonym list and a collection of previously-curated documents.) In addition, it might be beneficial to improve on the quality of the information sources used – for example, to use NER components trained on a more appropriate set of documents, to use domain-specific string similarity measures, or to use less noisy tagged historical data. It is also quite possible that performance would improve with larger collections of previously-curated documents; in general, one would expect the performance of this system to improve over time, as the corpus of labeled history data grows.

Our performance measures are *not *directly comparable to those used in the BioCreAtIvE workshop. In BioCreAtIvE, systems were evaluated by pooling all predicted abstract-protein matches across the test set, and computing the F-measure of this pool against a gold standard for the test set. All of the ranking systems proposed here can (and do) assign quite different absolute scores for each query, so there is no straightforward way to combine their predictions into a single pool. Indeed, one of the claims of this paper is that for an interactive, semi-automated curator's assistant, aggregating F-measure over many abstracts is not an appropriate performance measure: instead it is better to average performance across many queries.

As a rough measure of relative performance, we computed the maximal F-measure (over any threshold) of each ranked list produced, and then averaged these measures over all queries. This "Average Maximum F-measure" computation supports the claim that the graph-based method outperforms both of the NER systems on which it is based. The average maximum Fl-measure of the graph-based system with learning is 0.755.

Three of the eight BioCreAtIvE participants achieved aggregate F1 scores above this. Two of these participants [26,27] made use of ProMiner, a system that incorporates several domain-specific engineered components, including rule-based synonym processing and extra resources like the BioMedical Abbreviation Server and a cellular process vocabulary. The best F-measure performance of a ProMiner-derived system was 0.791. The third BioCreAtIvE system to achieve F-measure scores above 0.755 was based on using a soft match against the synonym list, followed by a filtering phase in which soft matches are tested with a classifier learned from the training-data abstracts [28]. This system obtained an aggregate F-measure performance of 0.758, and like our system, requires no special components that must be engineered for a new domain. However, while the soft-match system achieved recall of 0.90 or higher on the BioCreAtIvE *yeast *and *fly *corpora, the recall was only 0.79 for the mouse corpora. This bounds the recall of their system. Another result published recently [29] achieved a comparable F-measure score of 0.75 on the mouse dataset using soft-matching to available dictionaries, and applying a disambiguation heuristic. The recall of their system was 0.73.

## Conclusion

We have proposed a method for solving the "geneId finding" problem using easily-available resources, and no problem-specific engineered components. Specifically, the suggested method requires only a dictionary of protein synonyms; one or more inexact NER systems, trained on rather different corpora; generic, problem-independent soft-matching methods for strings; and a set of previously-curated documents that have been associated with protein identifiers mentioned in the documents.

In our proposed method, a graph is generated that combines the historical data and the outputs of multiple NER systems, and produces a ranked list of gene identifiers by traversing this graph with a stochastic approximation to a 10-step *lazy random walk*. This ranked list can then be re-ranked using a learned weight vector, if there is training data available in the form of documents paired with gene identifiers. This framework is general, as it can be readily used for many related tasks, like producing gene identifiers for a particular protein name, or finding abstracts likely to contain a protein given a protein identifier.

We tested our method on the BioCreAtIvE datasets for mouse proteins – mouse being one model organism that was found to be difficult for previous researchers. The graph-based approach outperformed any of its component NER systems. On a 50-problem development set, the graph-based approach with learning outperforms the best-performing single NER-system by nearly 30%, as measured by mean average precision; also, the graph-based approach *without *learning outperforms the best-performing single NER-system by more than 16%. Compared to a plausible baseline method which uses the single NER system with the highest F-measure, the graph-based approach with learning improves performance on the 50-problem development set by nearly 80%, and on a larger, 250-abstract prospective test set, improves performance over this baseline by more than 93%.

Our system cannot be easily adapted to aggregate predictions from multiple abstracts; however, if the *aggregate *performance of previous systems across the 250-abstract test set can be taken as representative of their *average *performance on individual problems, then the performance of our system is comparable to the best previous systems applied to this dataset.

A central claim we make in this paper is that a ranked list of protein identifiers is more useful than a fixed set, as it is more suitable for semi-automatic curation. In absolute terms, the performance is probably satisfactory to aid in curation: for instance, on average, more than 84% of the protein identifiers that should be associated with an abstract are ranked in the top five. In practical terms, the usefulness of a real curator's assistant based on this sort of ranking scheme might well be dominated not by minor improvements in the ranking, but by user-interface issues (e.g., how to explain to a curator why a protein was suggested).

## Methods

### Datasets

The training data and development data are subsets of the BioCreAtIvE "devtest" set (abstract numbers 0–99 and 100–149 respectively). The historical data was called "training data" in the BioCreAtIvE publications, but we use the term "historical data": we did not use them as training data *per se*, because this data is clearly different in character from the test data.

To evaluate NER systems, the development-data abstracts were hand-annotated (by the first author) by marking up all gene-protein entity names.

### NER systems

We used two closely related NER systems in our experiments. Both were trained using an off-the-shelf machine learning system for NER called Minorthird [14] on the YAPEX corpora [15].

The YAPEX corpus consists of a training corpus of 99 MEDLINE abstracts and a testing corpus of 101 MEDLINE abstracts. These documents deal primarily with protein-protein interactions, and are annotated for gene-protein entities. They contain 1745 and 1966 entities, respectively.

To train the first NER system, we used version 8.6.3.15 of Minorthird. We used the default to-kenizer, the "Recommended.TokenFE" feature extractor, and Minorthird's implementation of a voted-perceptron based training scheme for HMMs due to Collins [16]. As we configured this learner, NER is reduced to the problem of classifying each token as the *beginning, end*, or *continuation *of a multi-token protein name; as the *unique *token in a one-token-long protein name; or as *outside *any protein name. Thus, the learning task is to find a function that maps a sequence of tokens **x **to a corresponding sequence of labels **y**, where each *y*_*i *_is one of the five classes listed above. The annotated text is tokenized and preprocessed to produce a set of (**x**, **y**) pairs for the learner. The learner also uses a *local feature function ***f **which maps a pair (**x**, **y**) and an index *i *to a vector of features **f**(*i*, **x**, **y**). It is required that each component *f*^*k *^of **f **is Markovian, i.e., that it can be defined as

*f*^*k*^(*i*, **x**, **y**) = *f*^*k*^(*g*^*k*^(*i*, **x**), *y*_*i*_, *y*_*i*-1_)

where *g*^*k *^is arbitrary. Most of the *f*^*k*^'s that we used were indicator functions that look for some combination of properties that must hold for *y*_*i*_, *x*_*i*_, and/or *x*_*i *± *d *_for some small *d*. For instance, one possible such feature function would be

f42(i,x,y)={1if yi=end and xi+1="mRNA"0else
 MathType@MTEF@5@5@+=feaafiart1ev1aaatCvAUfKttLearuWrP9MDH5MBPbIqV92AaeXatLxBI9gBaebbnrfifHhDYfgasaacH8akY=wiFfYdH8Gipec8Eeeu0xXdbba9frFj0=OqFfea0dXdd9vqai=hGuQ8kuc9pgc9s8qqaq=dirpe0xb9q8qiLsFr0=vr0=vr0dc8meaabaqaciaacaGaaeqabaqabeGadaaakeaacqWGMbGzdaahaaWcbeqaaiabisda0iabikdaYaaakiabcIcaOiabdMgaPjabcYcaSGqabiab=Hha4jabcYcaSiab=Lha5jabcMcaPiabg2da9maaceqabaqbaeaabiGaaaqaaiabigdaXaqaaiabbMgaPjabbAgaMjabbccaGiabdMha5naaBaaaleaacqWGPbqAaeqaaOGaeyypa0JaemyzauMaemOBa4MaemizaqMaeeiiaaIaeeyyaeMaeeOBa4MaeeizaqMaeeiiaaIaemiEaG3aaSbaaSqaaiabdMgaPjabgUcaRiabigdaXaqabaGccqGH9aqpcqGGIaGicqqGTbqBcqqGsbGucqqGobGtcqqGbbqqcqGGIaGiaeaacqaIWaamaeaacqqGLbqzcqqGSbaBcqqGZbWCcqqGLbqzaaaacaGL7baaaaa@5EA8@

Defining **F**(**x**, **y**) = ∑i|x|f(i,x,y)
 MathType@MTEF@5@5@+=feaafiart1ev1aaatCvAUfKttLearuWrP9MDH5MBPbIqV92AaeXatLxBI9gBaebbnrfifHhDYfgasaacH8akY=wiFfYdH8Gipec8Eeeu0xXdbba9frFj0=OqFfea0dXdd9vqai=hGuQ8kuc9pgc9s8qqaq=dirpe0xb9q8qiLsFr0=vr0=vr0dc8meaabaqaciaacaGaaeqabaqabeGadaaakeaadaaeWaqaaGqabiab=zgaMjabcIcaOiabdMgaPjabcYcaSiab=Hha4jabcYcaSiab=Lha5jabcMcaPaWcbaGaemyAaKgabaGaeiiFaWNae8hEaGNaeiiFaWhaniabggHiLdaaaa@3D92@ and letting **W **be a weight vector over the components of **F**, we can now succinctly describe the learning method, the goal of which is to find a **W **that leads to the globally best overall performance. This "best" **W **is found by repeatedly updating **W **to improve the quality of the Viterbi decoding on a particular example (**x**_*t*_, **y**_*t*_). Specifically, Collins' algorithm starts with **W**_0 _= **0**, and looks at each example in turn. After the *t*-th example **x**_*t*_, **y**_*t*_, the Viterbi sequence y^
 MathType@MTEF@5@5@+=feaafiart1ev1aaatCvAUfKttLearuWrP9MDH5MBPbIqV92AaeXatLxBI9gBaebbnrfifHhDYfgasaacH8akY=wiFfYdH8Gipec8Eeeu0xXdbba9frFj0=OqFfea0dXdd9vqai=hGuQ8kuc9pgc9s8qqaq=dirpe0xb9q8qiLsFr0=vr0=vr0dc8meaabaqaciaacaGaaeqabaqabeGadaaakeaaieqacuWF5bqEgaqcaaaa@2E3D@_*t *_= *argmax*_**y **_**W**_*t*_·**F**(**x**, **y**) is computed, and **W**_*t *_is replaced with

**W**_*t*+1 _= **W**_*t *_+ **F**(**x**_*t*_, **y**_*t*_) - **F**(**x**_*t*_, y^
 MathType@MTEF@5@5@+=feaafiart1ev1aaatCvAUfKttLearuWrP9MDH5MBPbIqV92AaeXatLxBI9gBaebbnrfifHhDYfgasaacH8akY=wiFfYdH8Gipec8Eeeu0xXdbba9frFj0=OqFfea0dXdd9vqai=hGuQ8kuc9pgc9s8qqaq=dirpe0xb9q8qiLsFr0=vr0=vr0dc8meaabaqaciaacaGaaeqabaqabeGadaaakeaaieqacuWF5bqEgaqcaaaa@2E3D@_*t*_)     (1)

After training, one takes as the final learned weight vector **W **the average value of **W**_*t *_over all time steps *t*.

We configured the algorithm to make 20 passes over the training data.

Unlike the case for simple classifiers, it is non-trivial to modify a sequential classifier to improve recall. We use the following scheme to trade recall for precision [19]. In the likely-protein extractor, there is one particular feature feature *f*^0 ^which is defined as follows:

f0(i,x,y)={1if yi=outside0else
 MathType@MTEF@5@5@+=feaafiart1ev1aaatCvAUfKttLearuWrP9MDH5MBPbIqV92AaeXatLxBI9gBaebbnrfifHhDYfgasaacH8akY=wiFfYdH8Gipec8Eeeu0xXdbba9frFj0=OqFfea0dXdd9vqai=hGuQ8kuc9pgc9s8qqaq=dirpe0xb9q8qiLsFr0=vr0=vr0dc8meaabaqaciaacaGaaeqabaqabeGadaaakeaacqWGMbGzdaahaaWcbeqaaiabicdaWaaakiabcIcaOiabdMgaPjabcYcaSGqabiab=Hha4jabcYcaSiab=Lha5jabcMcaPiabg2da9maaceqabaqbaeaabiGaaaqaaiabigdaXaqaaiabbMgaPjabbAgaMjabbccaGiabdMha5naaBaaaleaacqWGPbqAaeqaaOGaeyypa0Jaem4Ba8MaemyDauNaemiDaqNaem4CamNaemyAaKMaemizaqMaemyzaugabaGaeGimaadabaGaeeyzauMaeeiBaWMaee4CamNaeeyzaugaaaGaay5Eaaaaaa@51A3@

As it turns out, voted-perceptron NER systems are quite sensitive to the value of *w*^0^, the weight in **W **assigned to *f*^0^. Let *P *and *R *be precision and recall, and define *the F*_*β *_*measure *as

Fβ=(β2+1)PRβ2P+R
 MathType@MTEF@5@5@+=feaafiart1ev1aaatCvAUfKttLearuWrP9MDH5MBPbIqV92AaeXatLxBI9gBaebbnrfifHhDYfgasaacH8akY=wiFfYdH8Gipec8Eeeu0xXdbba9frFj0=OqFfea0dXdd9vqai=hGuQ8kuc9pgc9s8qqaq=dirpe0xb9q8qiLsFr0=vr0=vr0dc8meaabaqaciaacaGaaeqabaqabeGadaaakeaacqWGgbGrdaWgaaWcbaacciGae8NSdigabeaakiabg2da9maalaaabaGaeiikaGIae8NSdi2aaWbaaSqabeaacqaIYaGmaaGccqGHRaWkcqaIXaqmcqGGPaqkcqWGqbaucqWGsbGuaeaacqWFYoGydaahaaWcbeqaaiabikdaYaaakiabdcfaqjabgUcaRiabdkfasbaaaaa@3F51@

It is common in information extraction research to use the F1-measure, a specialization of the more general *F*_*β *_measure. The F1-measure assigns *β *= 1, thus assuming equal importance to precision and recall. In general, *β *> 1 assigns higher importance to recall, and vice versa. We adjusted *w*^0 ^to optimize *F*_3 _on the YAPEX test set, using a Gauss-Newton line search in which the objective function is iteratively approximated by quadratics.

### Building the graph

Minorthird's NER system store their output in a particular data structure, and in incorporating the NER system's output, we found it easiest to construct an analog of that data structure in the graph. Rather than linking a document directly to entities found by the NER system, we constructed a *labels *node (which corresponds to Minorthird's "TextLabels" data structure). A document is linked to a *labels *node, and the *labels *node is then linked to the extracted entity strings. For each NER component *c*, the *labels *node is also linked to a special *NER-component *node, which is in turn linked to the entities extracted by that particular component. This particular choice of representation was largely a matter of programming convenience.

### Approximate name matching

The *soft TFIDF *[21] method was implemented using the SecondString open-source software package [30]. SoftTFIDF is based on *TFIDF similarity; *to compute this distance metric, a string *s *is first broken into a set *T*_*s *_of "tokens" (i.e., words). Each token *w *in *T*_*s *_is given a numeric weight *weight' *(*w*, *s*), based on the formula *weight' *(*w, s*) = log(TF_*w*,*s *_+ 1)·log(IDF_*w*_), where TF_*w*,*s *_is the frequency of word *w *in *s*, and IDF_*w *_is the inverse of the number of gene synonyms that contain *w*. Let

weight(w,s)=weight′(w,s)∑w′∈Trweight′(w′,r)2     (2)
 MathType@MTEF@5@5@+=feaafiart1ev1aaatCvAUfKttLearuWrP9MDH5MBPbIqV92AaeXatLxBI9gBaebbnrfifHhDYfgasaacH8akY=wiFfYdH8Gipec8Eeeu0xXdbba9frFj0=OqFfea0dXdd9vqai=hGuQ8kuc9pgc9s8qqaq=dirpe0xb9q8qiLsFr0=vr0=vr0dc8meaabaqaciaacaGaaeqabaqabeGadaaakeaacqWG3bWDcqWGLbqzcqWGPbqAcqWGNbWzcqWGObaAcqWG0baDcqGGOaakcqWG3bWDcqGGSaalcqWGZbWCcqGGPaqkcqGH9aqpdaWcaaqaaiabdEha3jabdwgaLjabdMgaPjabdEgaNjabdIgaOjqbdsha0zaafaGaeiikaGIaem4DaCNaeiilaWIaem4CamNaeiykaKcabaWaaOaaaeaadaaeqaqaaiabdEha3jabdwgaLjabdMgaPjabdEgaNjabdIgaOjqbdsha0zaafaGaeiikaGIafm4DaCNbauaacqGGSaalcqWGYbGCcqGGPaqkdaahaaWcbeqaaiabikdaYaaaaeaacuWG3bWDgaqbaiabgIGiolabdsfaunaaBaaameaacqWGYbGCaeqaaaWcbeqdcqGHris5aaWcbeaaaaGccaWLjaGaaCzcamaabmaabaGaeGOmaidacaGLOaGaayzkaaaaaa@63D6@

We now define the distance between the two words sets *T*_*r *_and *T*_*s *_as

TFIDF(Tr,Ts)=∑w∈Tr∩Tsweight(w,r)⋅weight(w,s)
 MathType@MTEF@5@5@+=feaafiart1ev1aaatCvAUfKttLearuWrP9MDH5MBPbIqV92AaeXatLxBI9gBaebbnrfifHhDYfgasaacH8akY=wiFfYdH8Gipec8Eeeu0xXdbba9frFj0=OqFfea0dXdd9vqai=hGuQ8kuc9pgc9s8qqaq=dirpe0xb9q8qiLsFr0=vr0=vr0dc8meaabaqaciaacaGaaeqabaqabeGadaaakeaajugqbiabdsfaujabdAeagjabdMeajjabdseaejabdAeagjabcIcaOiabdsfauPWaaSbaaSqaaiabdkhaYbqabaGccqGGSaalcqWGubavdaWgaaWcbaGaem4CamhabeaakiabcMcaPiabg2da9maaqafabaGaem4DaCNaemyzauMaemyAaKMaem4zaCMaemiAaGMaemiDaqNaeiikaGIaem4DaCNaeiilaWIaemOCaiNaeiykaKIaeyyXICTaem4DaCNaemyzauMaemyAaKMaem4zaCMaemiAaGMaemiDaqNaeiikaGIaem4DaCNaeiilaWIaem4CamNaeiykaKcaleaacqWG3bWDcqGHiiIZcqWGubavdaWgaaadbaGaemOCaihabeaaliabgMIihlabdsfaunaaBaaameaacqWGZbWCaeqaaaWcbeqdcqGHris5aaaa@665C@

Two advantages of TFIDF over edit-distance based similarity metrics are: first, token order is not important, so "B-chain of lactate dehydrogenase", "lactate dehydrogenase-B" are considered similar; and second, common but uninformative words like "chain" do not greatly affect similarity.

*SoftTFIDF *[21,31] is a "softer" version of TFIDF, in which similar tokens are considered as well as tokens in *T*_*s*_∩*T*_*r*_. Letting *sim' *be a secondary similarity function that is suited to comparing single tokens, we define

SoftTFIDF(Tr,Ts)=∑w∑w′weight(w,r)⋅weight(w′,s)⋅sim′(w,w′)
 MathType@MTEF@5@5@+=feaafiart1ev1aaatCvAUfKttLearuWrP9MDH5MBPbIqV92AaeXatLxBI9gBaebbnrfifHhDYfgasaacH8akY=wiFfYdH8Gipec8Eeeu0xXdbba9frFj0=OqFfea0dXdd9vqai=hGuQ8kuc9pgc9s8qqaq=dirpe0xb9q8qiLsFr0=vr0=vr0dc8meaabaqaciaacaGaaeqabaqabeGadaaakeaacqWGtbWucqWGVbWBcqWGMbGzcqWG0baDcqWGubavcqWGgbGrcqWGjbqscqWGebarcqWGgbGrcqGGOaakcqWGubavdaWgaaWcbaGaemOCaihabeaakiabcYcaSiabdsfaunaaBaaaleaacqWGZbWCaeqaaOGaeiykaKIaeyypa0ZaaabuaeaadaaeqbqaaiabdEha3jabdwgaLjabdMgaPjabdEgaNjabdIgaOjabdsha0jabcIcaOiabdEha3jabcYcaSiabdkhaYjabcMcaPiabgwSixlabdEha3jabdwgaLjabdMgaPjabdEgaNjabdIgaOjabdsha0jabcIcaOiqbdEha3zaafaGaeiilaWIaem4CamNaeiykaKIaeyyXICTaem4CamNaemyAaKMafmyBa0MbauaacqGGOaakcqWG3bWDcqGGSaalcuWG3bWDgaqbaiabcMcaPaWcbaGafm4DaCNbauaaaeqaniabggHiLdaaleaacqWG3bWDaeqaniabggHiLdaaaa@71F0@

The secondary similarity metric we used was the *Jaro-Winkler *metric [21]. Given strings *s *= *a*_1_...*a*_*K *_and *t *= *b*_1_...*b*_*L*_, define a character *a*_*i *_in *s *to be *common with t *there is a *b*_*j *_= *a*_*i *_in *t *such that *i *- *H *≤ *j *≤ *i *+ *H*, where H=min⁡(|s|,|t|)2
 MathType@MTEF@5@5@+=feaafiart1ev1aaatCvAUfKttLearuWrP9MDH5MBPbIqV92AaeXatLxBI9gBaebbnrfifHhDYfgasaacH8akY=wiFfYdH8Gipec8Eeeu0xXdbba9frFj0=OqFfea0dXdd9vqai=hGuQ8kuc9pgc9s8qqaq=dirpe0xb9q8qiLsFr0=vr0=vr0dc8meaabaqaciaacaGaaeqabaqabeGadaaakeaacqWGibascqGH9aqpdaWcaaqaaiGbc2gaTjabcMgaPjabc6gaUjabcIcaOiabcYha8jabdohaZjabcYha8jabcYcaSiabcYha8jabdsha0jabcYha8jabcMcaPaqaaiabikdaYaaaaaa@3F61@. Let *s' *= a′1
 MathType@MTEF@5@5@+=feaafiart1ev1aaatCvAUfKttLearuWrP9MDH5MBPbIqV92AaeXatLxBI9gBaebbnrfifHhDYfgasaacH8akY=wiFfYdH8Gipec8Eeeu0xXdbba9frFj0=OqFfea0dXdd9vqai=hGuQ8kuc9pgc9s8qqaq=dirpe0xb9q8qiLsFr0=vr0=vr0dc8meaabaqaciaacaGaaeqabaqabeGadaaakeaacuWGHbqygaqbamaaBaaaleaacqaIXaqmaeqaaaaa@2F1F@...a′K′
 MathType@MTEF@5@5@+=feaafiart1ev1aaatCvAUfKttLearuWrP9MDH5MBPbIqV92AaeXatLxBI9gBaebbnrfifHhDYfgasaacH8akY=wiFfYdH8Gipec8Eeeu0xXdbba9frFj0=OqFfea0dXdd9vqai=hGuQ8kuc9pgc9s8qqaq=dirpe0xb9q8qiLsFr0=vr0=vr0dc8meaabaqaciaacaGaaeqabaqabeGadaaakeaacuWGHbqygaqbamaaBaaaleaacuWGlbWsgaqbaaqabaaaaa@2F5A@, be the characters in *s *which are common with *t*, in the same order they appear in *s*, and let *t' *= b′1
 MathType@MTEF@5@5@+=feaafiart1ev1aaatCvAUfKttLearuWrP9MDH5MBPbIqV92AaeXatLxBI9gBaebbnrfifHhDYfgasaacH8akY=wiFfYdH8Gipec8Eeeu0xXdbba9frFj0=OqFfea0dXdd9vqai=hGuQ8kuc9pgc9s8qqaq=dirpe0xb9q8qiLsFr0=vr0=vr0dc8meaabaqaciaacaGaaeqabaqabeGadaaakeaacuWGIbGygaqbamaaBaaaleaacqaIXaqmaeqaaaaa@2F21@...b′L′
 MathType@MTEF@5@5@+=feaafiart1ev1aaatCvAUfKttLearuWrP9MDH5MBPbIqV92AaeXatLxBI9gBaebbnrfifHhDYfgasaacH8akY=wiFfYdH8Gipec8Eeeu0xXdbba9frFj0=OqFfea0dXdd9vqai=hGuQ8kuc9pgc9s8qqaq=dirpe0xb9q8qiLsFr0=vr0=vr0dc8meaabaqaciaacaGaaeqabaqabeGadaaakeaacuWGIbGygaqbamaaBaaaleaacuWGmbatgaqbaaqabaaaaa@2F5E@ be analogous, and define a *transposition for s', t' *to be a position *i *such that a′i
 MathType@MTEF@5@5@+=feaafiart1ev1aaatCvAUfKttLearuWrP9MDH5MBPbIqV92AaeXatLxBI9gBaebbnrfifHhDYfgasaacH8akY=wiFfYdH8Gipec8Eeeu0xXdbba9frFj0=OqFfea0dXdd9vqai=hGuQ8kuc9pgc9s8qqaq=dirpe0xb9q8qiLsFr0=vr0=vr0dc8meaabaqaciaacaGaaeqabaqabeGadaaakeaacuWGHbqygaqbamaaBaaaleaacqWGPbqAaeqaaaaa@2F8A@ ≠ b′i
 MathType@MTEF@5@5@+=feaafiart1ev1aaatCvAUfKttLearuWrP9MDH5MBPbIqV92AaeXatLxBI9gBaebbnrfifHhDYfgasaacH8akY=wiFfYdH8Gipec8Eeeu0xXdbba9frFj0=OqFfea0dXdd9vqai=hGuQ8kuc9pgc9s8qqaq=dirpe0xb9q8qiLsFr0=vr0=vr0dc8meaabaqaciaacaGaaeqabaqabeGadaaakeaacuWGIbGygaqbamaaBaaaleaacqWGPbqAaeqaaaaa@2F8C@. Let *H*_*s'*,*t' *_be half the number of transpositions for *s' *and *t'*. The Jaro metric for *s *and *t *is

Jaro(s,t)=13⋅(|s′||s|+|t′||t|+|s′|−Hs′,t′|s′|)
 MathType@MTEF@5@5@+=feaafiart1ev1aaatCvAUfKttLearuWrP9MDH5MBPbIqV92AaeXatLxBI9gBaebbnrfifHhDYfgasaacH8akY=wiFfYdH8Gipec8Eeeu0xXdbba9frFj0=OqFfea0dXdd9vqai=hGuQ8kuc9pgc9s8qqaq=dirpe0xb9q8qiLsFr0=vr0=vr0dc8meaabaqaciaacaGaaeqabaqabeGadaaakeaacqWGkbGscqWGHbqycqWGYbGCcqWGVbWBcqGGOaakcqWGZbWCcqGGSaalcqWG0baDcqGGPaqkcqGH9aqpdaWcaaqaaiabigdaXaqaaiabiodaZaaacqGHflY1daqadaqaamaalaaabaGaeiiFaWNafm4CamNbauaacqGG8baFaeaacqGG8baFcqWGZbWCcqGG8baFaaGaey4kaSYaaSaaaeaacqGG8baFcuWG0baDgaqbaiabcYha8bqaaiabcYha8jabdsha0jabcYha8baacqGHRaWkdaWcaaqaaiabcYha8jqbdohaZzaafaGaeiiFaWNaeyOeI0IaemisaG0aaSbaaSqaaiqbdohaZzaafaGaeiilaWIafmiDaqNbauaaaeqaaaGcbaGaeiiFaWNafm4CamNbauaacqGG8baFaaaacaGLOaGaayzkaaaaaa@60FD@

and finally, letting *P' *= max(P, 4),

Jaro−Winkler(s,t)=Jaro(s,t)+P′10⋅(1−Jaro(s,t))
 MathType@MTEF@5@5@+=feaafiart1ev1aaatCvAUfKttLearuWrP9MDH5MBPbIqV92AaeXatLxBI9gBaebbnrfifHhDYfgasaacH8akY=wiFfYdH8Gipec8Eeeu0xXdbba9frFj0=OqFfea0dXdd9vqai=hGuQ8kuc9pgc9s8qqaq=dirpe0xb9q8qiLsFr0=vr0=vr0dc8meaabaqaciaacaGaaeqabaqabeGadaaakeaacqWGkbGscqWGHbqycqWGYbGCcqWGVbWBcqGHsislcqWGxbWvcqWGPbqAcqWGUbGBcqWGRbWAcqWGSbaBcqWGLbqzcqWGYbGCcqGGOaakcqWGZbWCcqGGSaalcqWG0baDcqGGPaqkcqGH9aqpcqWGkbGscqWGHbqycqWGYbGCcqWGVbWBcqGGOaakcqWGZbWCcqGGSaalcqWG0baDcqGGPaqkcqGHRaWkdaWcaaqaaiqbdcfaqzaafaaabaGaeGymaeJaeGimaadaaiabgwSixlabcIcaOiabigdaXiabgkHiTiabdQeakjabdggaHjabdkhaYjabd+gaVjabcIcaOiabdohaZjabcYcaSiabdsha0jabcMcaPiabcMcaPaaa@61FE@

### Combining NER and soft matching

To reduce the cost of repeatedly applying the softTFIDF algorithm in matching all possible entity pairs, we constructed an inverted index for each token that occured in the gene synonym list, and only computed similarities between a string *s *and those strings *t *that contain at least one token in *s*. (For the purpose of this particular computation, a token is any maximal subsequence of alphanumeric characters: thus "Int-3" would be two tokens, not one.)

### Graph search for ranking

#### Formal details

Formally, a graph *G *consists of a set of nodes, and a set of labeled directed edges. Nodes will be denoted by letters like *x*, *y*, or *z*, and we will denote an edge from *x *to *y *with label ℓ as x→ℓy
 MathType@MTEF@5@5@+=feaafiart1ev1aaatCvAUfKttLearuWrP9MDH5MBPbIqV92AaeXatLxBI9gBaebbnrfifHhDYfgasaacH8akY=wiFfYdH8Gipec8Eeeu0xXdbba9frFj0=OqFfea0dXdd9vqai=hGuQ8kuc9pgc9s8qqaq=dirpe0xb9q8qiLsFr0=vr0=vr0dc8meaabaqaciaacaGaaeqabaqabeGadaaakeaacqWG4baEdaGdKaWcbaGaeS4eHWgabeGccaGLsgcacqWG5bqEaaa@3269@. Every node *x *has a *type*. Each node represents some unique entity, and each edge x→ℓy
 MathType@MTEF@5@5@+=feaafiart1ev1aaatCvAUfKttLearuWrP9MDH5MBPbIqV92AaeXatLxBI9gBaebbnrfifHhDYfgasaacH8akY=wiFfYdH8Gipec8Eeeu0xXdbba9frFj0=OqFfea0dXdd9vqai=hGuQ8kuc9pgc9s8qqaq=dirpe0xb9q8qiLsFr0=vr0=vr0dc8meaabaqaciaacaGaaeqabaqabeGadaaakeaacqWG4baEdaGdKaWcbaGaeS4eHWgabeGccaGLsgcacqWG5bqEaaa@3269@ asserts that some binary relation ℓ(*x*, *y*) holds. We will assume, for convenience, that there are no edges from a node to itself. Multiple relations can hold between any particular pair of nodes types: for instance, it could be that x→ℓy
 MathType@MTEF@5@5@+=feaafiart1ev1aaatCvAUfKttLearuWrP9MDH5MBPbIqV92AaeXatLxBI9gBaebbnrfifHhDYfgasaacH8akY=wiFfYdH8Gipec8Eeeu0xXdbba9frFj0=OqFfea0dXdd9vqai=hGuQ8kuc9pgc9s8qqaq=dirpe0xb9q8qiLsFr0=vr0=vr0dc8meaabaqaciaacaGaaeqabaqabeGadaaakeaacqWG4baEdaGdKaWcbaGaeS4eHWgabeGccaGLsgcacqWG5bqEaaa@3269@ or x→ℓ′y
 MathType@MTEF@5@5@+=feaafiart1ev1aaatCvAUfKttLearuWrP9MDH5MBPbIqV92AaeXatLxBI9gBaebbnrfifHhDYfgasaacH8akY=wiFfYdH8Gipec8Eeeu0xXdbba9frFj0=OqFfea0dXdd9vqai=hGuQ8kuc9pgc9s8qqaq=dirpe0xb9q8qiLsFr0=vr0=vr0dc8meaabaqaciaacaGaaeqabaqabeGadaaakeaacqWG4baEdaGdKaWcbaGafS4eHWMbauaaaeqakiaawkziaiabdMha5baa@3275@, where ℓ ≠ ℓ'. Also, edges need not denote functional relations: i.e., for a given *x *and ℓ, there may be many distinct nodes *y *such that x→ℓy
 MathType@MTEF@5@5@+=feaafiart1ev1aaatCvAUfKttLearuWrP9MDH5MBPbIqV92AaeXatLxBI9gBaebbnrfifHhDYfgasaacH8akY=wiFfYdH8Gipec8Eeeu0xXdbba9frFj0=OqFfea0dXdd9vqai=hGuQ8kuc9pgc9s8qqaq=dirpe0xb9q8qiLsFr0=vr0=vr0dc8meaabaqaciaacaGaaeqabaqabeGadaaakeaacqWG4baEdaGdKaWcbaGaeS4eHWgabeGccaGLsgcacqWG5bqEaaa@3269@.

Similarity between two nodes is defined by a *lazy walk process*. To walk away from a node *x*, one first picks an edge label ℓ; then, given ℓ, one picks a node *y *such that x→ℓy
 MathType@MTEF@5@5@+=feaafiart1ev1aaatCvAUfKttLearuWrP9MDH5MBPbIqV92AaeXatLxBI9gBaebbnrfifHhDYfgasaacH8akY=wiFfYdH8Gipec8Eeeu0xXdbba9frFj0=OqFfea0dXdd9vqai=hGuQ8kuc9pgc9s8qqaq=dirpe0xb9q8qiLsFr0=vr0=vr0dc8meaabaqaciaacaGaaeqabaqabeGadaaakeaacqWG4baEdaGdKaWcbaGaeS4eHWgabeGccaGLsgcacqWG5bqEaaa@3269@. We assume that the probability of picking the label ℓ is uniform over all label types *L*(*x*) that label edges leaving *x *– i.e., that Pr⁡(ℓ|x)=1|L(x)|
 MathType@MTEF@5@5@+=feaafiart1ev1aaatCvAUfKttLearuWrP9MDH5MBPbIqV92AaeXatLxBI9gBaebbnrfifHhDYfgasaacH8akY=wiFfYdH8Gipec8Eeeu0xXdbba9frFj0=OqFfea0dXdd9vqai=hGuQ8kuc9pgc9s8qqaq=dirpe0xb9q8qiLsFr0=vr0=vr0dc8meaabaqaciaacaGaaeqabaqabeGadaaakeaacyGGqbaucqGGYbGCcqGGOaakcqWItecBcqGG8baFcqWG4baEcqGGPaqkcqGH9aqpdaWcaaqaaiabigdaXaqaaiabcYha8jabdYeamjabcIcaOiabdIha4jabcMcaPiabcYha8baaaaa@3E70@ where

L(x)={ℓ:∃y.x→ℓy}
 MathType@MTEF@5@5@+=feaafiart1ev1aaatCvAUfKttLearuWrP9MDH5MBPbIqV92AaeXatLxBI9gBaebbnrfifHhDYfgasaacH8akY=wiFfYdH8Gipec8Eeeu0xXdbba9frFj0=OqFfea0dXdd9vqai=hGuQ8kuc9pgc9s8qqaq=dirpe0xb9q8qiLsFr0=vr0=vr0dc8meaabaqaciaacaGaaeqabaqabeGadaaakeaacqWGmbatcqGGOaakcqWG4baEcqGGPaqkcqGH9aqpcqGG7bWEcqWItecBcqGG6aGocqGHdicjcqWG5bqEcqGGUaGlcqWG4baEdaGdKaWcbaGaeS4eHWgabeGccaGLsgcacqWG5bqEcqGG9bqFaaa@401C@

For most node and edge types, after ℓ is picked, *y *is chosen uniformly from the set of all *y *such that x→ℓy
 MathType@MTEF@5@5@+=feaafiart1ev1aaatCvAUfKttLearuWrP9MDH5MBPbIqV92AaeXatLxBI9gBaebbnrfifHhDYfgasaacH8akY=wiFfYdH8Gipec8Eeeu0xXdbba9frFj0=OqFfea0dXdd9vqai=hGuQ8kuc9pgc9s8qqaq=dirpe0xb9q8qiLsFr0=vr0=vr0dc8meaabaqaciaacaGaaeqabaqabeGadaaakeaacqWG4baEdaGdKaWcbaGaeS4eHWgabeGccaGLsgcacqWG5bqEaaa@3269@. If we likewise define

Y(x,ℓ)={y:x→ℓy}
 MathType@MTEF@5@5@+=feaafiart1ev1aaatCvAUfKttLearuWrP9MDH5MBPbIqV92AaeXatLxBI9gBaebbnrfifHhDYfgasaacH8akY=wiFfYdH8Gipec8Eeeu0xXdbba9frFj0=OqFfea0dXdd9vqai=hGuQ8kuc9pgc9s8qqaq=dirpe0xb9q8qiLsFr0=vr0=vr0dc8meaabaqaciaacaGaaeqabaqabeGadaaakeaacqWGzbqwcqGGOaakcqWG4baEcqGGSaalcqWItecBcqGGPaqkcqGH9aqpcqGG7bWEcqWG5bqEcqGG6aGocqWG4baEdaGdKaWcbaGaeS4eHWgabeGccaGLsgcacqWG5bqEcqGG9bqFaaa@3F5D@

then we can also easily define this default weighting scheme as Pr(*y*|*x*, ℓ) = Pr⁡(y|x,ℓ)=1|Y(x,ℓ)|
 MathType@MTEF@5@5@+=feaafiart1ev1aaatCvAUfKttLearuWrP9MDH5MBPbIqV92AaeXatLxBI9gBaebbnrfifHhDYfgasaacH8akY=wiFfYdH8Gipec8Eeeu0xXdbba9frFj0=OqFfea0dXdd9vqai=hGuQ8kuc9pgc9s8qqaq=dirpe0xb9q8qiLsFr0=vr0=vr0dc8meaabaqaciaacaGaaeqabaqabeGadaaakeaacyGGqbaucqGGYbGCcqGGOaakcqWG5bqEcqGG8baFcqWG4baEcqGGSaalcqWItecBcqGGPaqkcqGH9aqpdaWcaaqaaiabigdaXaqaaiabcYha8jabdMfazjabcIcaOiabdIha4jabcYcaSiabloriSjabcMcaPiabcYha8baaaaa@42F6@. Two exceptions to this default scheme are edges of type ℓ = *hasTerm *and ℓ = *inFile*. As detailed below, we use an open-source full-text retrieval engine to implement these edge types. When given a term *x *as a search query, this engine retrieves files *y *that contain *x*, and also provides a similarity score for these files *y*, which is a slight variant of the TFIDF similarity of *y *to a one-term document containing *x*. We weight files *y *proportionally to this similarity score, and thus Pr(*y*|*x*, *inFile*) is non-uniform. For the edge type ℓ = *hasTerm*, we also weight terms *w *in a string (or file) proportionally to their TFIDF weight, as computed by Equation 2; in this context IDF_*w *_represents the inverse of the total number of *file *and *string *nodes that contain *w*.

Conceptually, the edge weights above define the probability of moving from a node *x *to some other node *y*. One can recursively define Q(x→=dz)
 MathType@MTEF@5@5@+=feaafiart1ev1aaatCvAUfKttLearuWrP9MDH5MBPbIqV92AaeXatLxBI9gBaebbnrfifHhDYfgasaacH8akY=wiFfYdH8Gipec8Eeeu0xXdbba9frFj0=OqFfea0dXdd9vqai=hGuQ8kuc9pgc9s8qqaq=dirpe0xb9q8qiLsFr0=vr0=vr0dc8meaabaqaciaacaGaaeqabaqabeGadaaakeaacqWGrbqucqGGOaakcqWG4baEdaGdKaWcbaGaeyypa0JaemizaqgabeGccaGLsgcacqWG6bGEcqGGPaqkaaa@366E@, the probability of moving from *x *to *z *in exactly *d *steps, as follows:

Q(x→=0z)=1Q(x→=dz)=∑y(∑ℓPr⁡(ℓ|x)⋅Pr⁡(y|x,ℓ))⋅Q(y→=d−1z)
 MathType@MTEF@5@5@+=feaafiart1ev1aaatCvAUfKttLearuWrP9MDH5MBPbIqV92AaeXatLxBI9gBaebbnrfifHhDYfgasaacH8akY=wiFfYdH8Gipec8Eeeu0xXdbba9frFj0=OqFfea0dXdd9vqai=hGuQ8kuc9pgc9s8qqaq=dirpe0xb9q8qiLsFr0=vr0=vr0dc8meaabaqaciaacaGaaeqabaqabeGadaaakeaafaqaaeGabaaabaGaemyuaeLaeiikaGIaemiEaG3aa4ajaSqaaiabg2da9iabicdaWaqabOGaayPKHaGaemOEaONaeiykaKIaeyypa0JaeGymaedabaGaemyuaeLaeiikaGIaemiEaG3aa4ajaSqaaiabg2da9iabdsgaKbqabOGaayPKHaGaemOEaONaeiykaKIaeyypa0ZaaabuaeaacqGGOaakdaaeqbqaaiGbccfaqjabckhaYjabcIcaOiabloriSjabcYha8jabdIha4jabcMcaPiabgwSixlGbccfaqjabckhaYjabcIcaOiabdMha5jabcYha8jabdIha4jabcYcaSiabloriSjabcMcaPiabcMcaPiabgwSixlabdgfarjabcIcaOiabdMha5naaoqcaleaacqGH9aqpcqWGKbazcqGHsislcqaIXaqmaeqakiaawkziaiabdQha6jabcMcaPaWcbaGaeS4eHWgabeqdcqGHris5aaWcbaGaemyEaKhabeqdcqGHris5aaaaaaa@6EED@

Given probability *γ *of stopping the walk, the probability *Q*(*z*|*x*) of stopping at *z *in an infinitely-long "lazy walk" from *x *is defined as

Q(z|x)=γ∑d=1∞(1−γ)dQ(x→=dz)
 MathType@MTEF@5@5@+=feaafiart1ev1aaatCvAUfKttLearuWrP9MDH5MBPbIqV92AaeXatLxBI9gBaebbnrfifHhDYfgasaacH8akY=wiFfYdH8Gipec8Eeeu0xXdbba9frFj0=OqFfea0dXdd9vqai=hGuQ8kuc9pgc9s8qqaq=dirpe0xb9q8qiLsFr0=vr0=vr0dc8meaabaqaciaacaGaaeqabaqabeGadaaakeaacqWGrbqucqGGOaakcqWG6bGEcqGG8baFcqWG4baEcqGGPaqkcqGH9aqpiiGacqWFZoWzdaaeWbqaaiabcIcaOiabigdaXiabgkHiTiab=n7aNjabcMcaPmaaCaaaleqabaGaemizaqgaaOGaemyuaeLaeiikaGIaemiEaG3aa4ajaSqaaiabg2da9iabdsgaKbqabOGaayPKHaGaemOEaONaeiykaKcaleaacqWGKbazcqGH9aqpcqaIXaqmaeaacqGHEisPa0GaeyyeIuoaaaa@4E28@

This can be approximated by limiting the summation to some maximal value *d*_*max*_.

The graph created from the mouse data is moderately large – over a million nodes – so we store it in secondary memory. We use the open-source search engine Lucene [32] to implement the *inFile *and *hasTerm *edges, and the open-source database package Sleepycat [33] to store the user-defined nodes and edges. For efficiency, we also limit to 50 the number of terms *y *accessible from a file *x *via *hasTerm*, and also limit to 50 the number of files *x *accessible from a term *y *via *inFile*.

We use *γ *= 12
 MathType@MTEF@5@5@+=feaafiart1ev1aaatCvAUfKttLearuWrP9MDH5MBPbIqV92AaeXatLxBI9gBaebbnrfifHhDYfgasaacH8akY=wiFfYdH8Gipec8Eeeu0xXdbba9frFj0=OqFfea0dXdd9vqai=hGuQ8kuc9pgc9s8qqaq=dirpe0xb9q8qiLsFr0=vr0=vr0dc8meaabaqaciaacaGaaeqabaqabeGadaaakeaadaWcaaqaaiabigdaXaqaaiabikdaYaaaaaa@2E9E@; and *d*_*max *_= 10 in our experiments. It is computationally infeasible to perform a full matrix multiplication for 10 iterations, so instead we use the sampling scheme shown in Table 3. If each node *x *in the graph has *b *or fewer neighbors – i.e., if ∀*x *∑_ℓ_|*Y *(*x*, ℓ)| <*b *– then the computation is reduced from time *O*(*Nbk*), where *N *is the number of nodes in the graph, to time *O*(*mbk*). We used *m *= 500 in our experiments. In practice computation time is completely dominated by disk access, so the most important optimization is to cache in memory information about *P*(*y*|*x*) for recently accessed nodes. On a commodity PC, the current implementation averages a little more than minute per search on the mouse development data. We believe that this can be sped up by a factor of 10 or more with more careful use of main memory.

**Table 3:** GeneId-Ranking With a Graph

(1) let *Q*_0 _be the probability distribution such that *Q*_0_(*x*) = 1
(2) for *d *= 1, ..., *d*_*max *_do
• let *Q*_*d*_(*x*) = 0 for all *x *
• for *i *= 1, ..., *m *do
- sample *x*_*i *_according to *Q*_*d*-1 _
- *Q*_*d*_(*x*_*i*_) = *γQ*_*d*-1 _(*x*_*i*_)
- for each edge label ℓ ∈ *L *(*x*)
* for each node *y *∈ *Y*(*x*, ℓ)
· let *q*_*xy *_= Pr(*y*|ℓ, *x*)·Pr(ℓ|*x*)
· increment *Q*_*d*_(*y*) by (1 - *γ*)*Q*_*d*-1 _(*x*_*i*_)*q*_*xy*_
(3) return Qdmax MathType@MTEF@5@5@+=feaafiart1ev1aaatCvAUfKttLearuWrP9MDH5MBPbIqV92AaeXatLxBI9gBaebbnrfifHhDYfgasaacH8akY=wiFfYdH8Gipec8Eeeu0xXdbba9frFj0=OqFfea0dXdd9vqai=hGuQ8kuc9pgc9s8qqaq=dirpe0xb9q8qiLsFr0=vr0=vr0dc8meaabaqaciaacaGaaeqabaqabeGadaaakeaacqWGrbqudaWgaaWcbaGaemizaq2aaSbaaWqaaGqaciab=1gaTjab=fgaHjab=Hha4bqabaaaleqaaaaa@33B2@ (*z*) as an approximation to *Q*(*z*|*x*)

An efficient approximation algorithm for computing *Q*(*z*|*x*), given transition probabilities Pr(*y*|*x*, ℓ) and Pr(ℓ|*x*).

### Learning to rank

Recall the definition of *Q*(*z|x*). We will use the same sort of recursive definition to build up a feature vector that describes a ranked item *z*. We will begin with a vector **f **of primitive feature functions that describe the individual edges in a graph. In our experiments, we constructed one such feature function for each possible combination of a source node type *T*(*x*)and destination node type *T*(*y*). The list of features used is given in Table 5.

**Table 5:** Features Used for Ranking

TestFilehasTerm→ MathType@MTEF@5@5@+=feaafiart1ev1aaatCvAUfKttLearuWrP9MDH5MBPbIqV92AaeXatLxBI9gBaebbnrfifHhDYfgasaacH8akY=wiFfYdH8Gipec8Eeeu0xXdbba9frFj0=OqFfea0dXdd9vqai=hGuQ8kuc9pgc9s8qqaq=dirpe0xb9q8qiLsFr0=vr0=vr0dc8meaabaqaciaacaGaaeqabaqabeGadaaakeaadaWfqaqaaiabbIgaOjabbggaHjabbohaZjabbsfaujabbwgaLjabbkhaYjabb2gaTbWcbaWaa4akaWqabeaaaSGaayPKHaaabeaaaaa@37DC@	EntityTypehasSpan→ MathType@MTEF@5@5@+=feaafiart1ev1aaatCvAUfKttLearuWrP9MDH5MBPbIqV92AaeXatLxBI9gBaebbnrfifHhDYfgasaacH8akY=wiFfYdH8Gipec8Eeeu0xXdbba9frFj0=OqFfea0dXdd9vqai=hGuQ8kuc9pgc9s8qqaq=dirpe0xb9q8qiLsFr0=vr0=vr0dc8meaabaqaciaacaGaaeqabaqabeGadaaakeaadaWfqaqaaGqaaiab=HgaOjab=fgaHjab=nhaZjab=nfatjab=bhaWjab=fgaHjab=5gaUbWcbaWaa4akaWqabeaaaSGaayPKHaaabeaaaaa@37CB@	StringhasPossibleProtein−1→ MathType@MTEF@5@5@+=feaafiart1ev1aaatCvAUfKttLearuWrP9MDH5MBPbIqV92AaeXatLxBI9gBaebbnrfifHhDYfgasaacH8akY=wiFfYdH8Gipec8Eeeu0xXdbba9frFj0=OqFfea0dXdd9vqai=hGuQ8kuc9pgc9s8qqaq=dirpe0xb9q8qiLsFr0=vr0=vr0dc8meaabaqaciaacaGaaeqabaqabeGadaaakeaadaWfqaqaaGqaaiab=HgaOjab=fgaHjab=nhaZjab=bfaqjab=9gaVjab=nhaZjab=nhaZjab=LgaPjab=jgaIjab=XgaSjab=vgaLjab=bfaqjab=jhaYjab=9gaVjab=rha0jab=vgaLjab=LgaPjab=5gaUnaaCaaaleqabaaccaGae4NeI0Iae8xmaedaaaqaamaaoGcameqabaaaliaawkziaaqabaaaaa@489A@
GeneSynsynonym−1→ MathType@MTEF@5@5@+=feaafiart1ev1aaatCvAUfKttLearuWrP9MDH5MBPbIqV92AaeXatLxBI9gBaebbnrfifHhDYfgasaacH8akY=wiFfYdH8Gipec8Eeeu0xXdbba9frFj0=OqFfea0dXdd9vqai=hGuQ8kuc9pgc9s8qqaq=dirpe0xb9q8qiLsFr0=vr0=vr0dc8meaabaqaciaacaGaaeqabaqabeGadaaakeaadaWfqaqaaiabbohaZjabbMha5jabb6gaUjabb+gaVjabb6gaUjabbMha5jabb2gaTnaaCaaaleqabaaccaGae8NeI0ccbaGae4xmaedaaaqaamaaoGcameqabaaaliaawkziaaqabaaaaa@3A6F@	LabelshasPossibleProtein→ MathType@MTEF@5@5@+=feaafiart1ev1aaatCvAUfKttLearuWrP9MDH5MBPbIqV92AaeXatLxBI9gBaebbnrfifHhDYfgasaacH8akY=wiFfYdH8Gipec8Eeeu0xXdbba9frFj0=OqFfea0dXdd9vqai=hGuQ8kuc9pgc9s8qqaq=dirpe0xb9q8qiLsFr0=vr0=vr0dc8meaabaqaciaacaGaaeqabaqabeGadaaakeaadaWfqaqaaiabbIgaOjabbggaHjabbohaZjabbcfaqjabb+gaVjabbohaZjabbohaZjabbMgaPjabbkgaIjabbYgaSjabbwgaLjabbcfaqjabbkhaYjabb+gaVjabbsha0jabbwgaLjabbMgaPjabb6gaUbWcbaWaa4akaWqabeaaaSGaayPKHaaabeaaaaa@46BD@	StringhasTerm→ MathType@MTEF@5@5@+=feaafiart1ev1aaatCvAUfKttLearuWrP9MDH5MBPbIqV92AaeXatLxBI9gBaebbnrfifHhDYfgasaacH8akY=wiFfYdH8Gipec8Eeeu0xXdbba9frFj0=OqFfea0dXdd9vqai=hGuQ8kuc9pgc9s8qqaq=dirpe0xb9q8qiLsFr0=vr0=vr0dc8meaabaqaciaacaGaaeqabaqabeGadaaakeaadaWfqaqaaiabbIgaOjabbggaHjabbohaZjabbsfaujabbwgaLjabbkhaYjabb2gaTbWcbaWaa4akaWqabeaaaSGaayPKHaaabeaaaaa@37DC@
TestFilepossibleProtNER→ MathType@MTEF@5@5@+=feaafiart1ev1aaatCvAUfKttLearuWrP9MDH5MBPbIqV92AaeXatLxBI9gBaebbnrfifHhDYfgasaacH8akY=wiFfYdH8Gipec8Eeeu0xXdbba9frFj0=OqFfea0dXdd9vqai=hGuQ8kuc9pgc9s8qqaq=dirpe0xb9q8qiLsFr0=vr0=vr0dc8meaabaqaciaacaGaaeqabaqabeGadaaakeaadaWfqaqaaiabbchaWjabb+gaVjabbohaZjabbohaZjabbMgaPjabbkgaIjabbYgaSjabbwgaLjabbcfaqjabbkhaYjabb+gaVjabbsha0jabb6eaojabbweafjabbkfasbWcbaWaa4akaWqabeaaaSGaayPKHaaabeaaaaa@4242@	GeneIdhasGene−1→ MathType@MTEF@5@5@+=feaafiart1ev1aaatCvAUfKttLearuWrP9MDH5MBPbIqV92AaeXatLxBI9gBaebbnrfifHhDYfgasaacH8akY=wiFfYdH8Gipec8Eeeu0xXdbba9frFj0=OqFfea0dXdd9vqai=hGuQ8kuc9pgc9s8qqaq=dirpe0xb9q8qiLsFr0=vr0=vr0dc8meaabaqaciaacaGaaeqabaqabeGadaaakeaadaWfqaqaaiabbIgaOjabbggaHjabbohaZjabbEeahjabbwgaLjabb6gaUjabbwgaLnaaCaaaleqabaaccaGae8NeI0ccbaGae4xmaedaaaqaamaaoGcameqabaaaliaawkziaaqabaaaaa@39AB@	TERMinFile→ MathType@MTEF@5@5@+=feaafiart1ev1aaatCvAUfKttLearuWrP9MDH5MBPbIqV92AaeXatLxBI9gBaebbnrfifHhDYfgasaacH8akY=wiFfYdH8Gipec8Eeeu0xXdbba9frFj0=OqFfea0dXdd9vqai=hGuQ8kuc9pgc9s8qqaq=dirpe0xb9q8qiLsFr0=vr0=vr0dc8meaabaqaciaacaGaaeqabaqabeGadaaakeaadaWfqaqaaiabbMgaPjabb6gaUjabbAeagjabbMgaPjabbYgaSjabbwgaLbWcbaWaa4akaWqabeaaaSGaayPKHaaabeaaaaa@365B@
TrainFilehasGene→ MathType@MTEF@5@5@+=feaafiart1ev1aaatCvAUfKttLearuWrP9MDH5MBPbIqV92AaeXatLxBI9gBaebbnrfifHhDYfgasaacH8akY=wiFfYdH8Gipec8Eeeu0xXdbba9frFj0=OqFfea0dXdd9vqai=hGuQ8kuc9pgc9s8qqaq=dirpe0xb9q8qiLsFr0=vr0=vr0dc8meaabaqaciaacaGaaeqabaqabeGadaaakeaadaWfqaqaaiabbIgaOjabbggaHjabbohaZjabbEeahjabbwgaLjabb6gaUjabbwgaLbWcbaWaa4akaWqabeaaaSGaayPKHaaabeaaaaa@37AA@	Labelsannotates→ MathType@MTEF@5@5@+=feaafiart1ev1aaatCvAUfKttLearuWrP9MDH5MBPbIqV92AaeXatLxBI9gBaebbnrfifHhDYfgasaacH8akY=wiFfYdH8Gipec8Eeeu0xXdbba9frFj0=OqFfea0dXdd9vqai=hGuQ8kuc9pgc9s8qqaq=dirpe0xb9q8qiLsFr0=vr0=vr0dc8meaabaqaciaacaGaaeqabaqabeGadaaakeaadaWfqaqaaiabbggaHjabb6gaUjabb6gaUjabb+gaVjabbsha0jabbggaHjabbsha0jabbwgaLjabbohaZbWcbaWaa4akaWqabeaaaSGaayPKHaaabeaaaaa@3ADC@	LabelshasLikelyProtein→ MathType@MTEF@5@5@+=feaafiart1ev1aaatCvAUfKttLearuWrP9MDH5MBPbIqV92AaeXatLxBI9gBaebbnrfifHhDYfgasaacH8akY=wiFfYdH8Gipec8Eeeu0xXdbba9frFj0=OqFfea0dXdd9vqai=hGuQ8kuc9pgc9s8qqaq=dirpe0xb9q8qiLsFr0=vr0=vr0dc8meaabaqaciaacaGaaeqabaqabeGadaaakeaadaWfqaqaaiabbIgaOjabbggaHjabbohaZjabbYeamjabbMgaPjabbUgaRjabbwgaLjabbYgaSjabbMha5jabbcfaqjabbkhaYjabb+gaVjabbsha0jabbwgaLjabbMgaPjabb6gaUbWcbaWaa4akaWqabeaaaSGaayPKHaaabeaaaaa@4401@
TrainFileannotates−1→ MathType@MTEF@5@5@+=feaafiart1ev1aaatCvAUfKttLearuWrP9MDH5MBPbIqV92AaeXatLxBI9gBaebbnrfifHhDYfgasaacH8akY=wiFfYdH8Gipec8Eeeu0xXdbba9frFj0=OqFfea0dXdd9vqai=hGuQ8kuc9pgc9s8qqaq=dirpe0xb9q8qiLsFr0=vr0=vr0dc8meaabaqaciaacaGaaeqabaqabeGadaaakeaadaWfqaqaaiabbggaHjabb6gaUjabb6gaUjabb+gaVjabbsha0jabbggaHjabbsha0jabbwgaLjabbohaZnaaCaaaleqabaaccaGae8NeI0ccbaGae4xmaedaaaqaamaaoGcameqabaaaliaawkziaaqabaaaaa@3CDD@	StringhasLikelyProtein−1→ MathType@MTEF@5@5@+=feaafiart1ev1aaatCvAUfKttLearuWrP9MDH5MBPbIqV92AaeXatLxBI9gBaebbnrfifHhDYfgasaacH8akY=wiFfYdH8Gipec8Eeeu0xXdbba9frFj0=OqFfea0dXdd9vqai=hGuQ8kuc9pgc9s8qqaq=dirpe0xb9q8qiLsFr0=vr0=vr0dc8meaabaqaciaacaGaaeqabaqabeGadaaakeaadaWfqaqaaiabbIgaOjabbggaHjabbohaZjabbYeamjabbMgaPjabbUgaRjabbwgaLjabbYgaSjabbMha5jabbcfaqjabbkhaYjabb+gaVjabbsha0jabbwgaLjabbMgaPjabb6gaUnaaCaaaleqabaaccaGae8NeI0ccbaGae4xmaedaaaqaamaaoGcameqabaaaliaawkziaaqabaaaaa@4602@	TestFilelikelyProtNER→ MathType@MTEF@5@5@+=feaafiart1ev1aaatCvAUfKttLearuWrP9MDH5MBPbIqV92AaeXatLxBI9gBaebbnrfifHhDYfgasaacH8akY=wiFfYdH8Gipec8Eeeu0xXdbba9frFj0=OqFfea0dXdd9vqai=hGuQ8kuc9pgc9s8qqaq=dirpe0xb9q8qiLsFr0=vr0=vr0dc8meaabaqaciaacaGaaeqabaqabeGadaaakeaadaWfqaqaaiabbYgaSjabbMgaPjabbUgaRjabbwgaLjabbYgaSjabbMha5jabbcfaqjabbkhaYjabb+gaVjabbsha0jabb6eaojabbweafjabbkfasbWcbaWaa4akaWqabeaaaSGaayPKHaaabeaaaaa@3F86@
TestFileannotates−1→ MathType@MTEF@5@5@+=feaafiart1ev1aaatCvAUfKttLearuWrP9MDH5MBPbIqV92AaeXatLxBI9gBaebbnrfifHhDYfgasaacH8akY=wiFfYdH8Gipec8Eeeu0xXdbba9frFj0=OqFfea0dXdd9vqai=hGuQ8kuc9pgc9s8qqaq=dirpe0xb9q8qiLsFr0=vr0=vr0dc8meaabaqaciaacaGaaeqabaqabeGadaaakeaadaWfqaqaaiabbggaHjabb6gaUjabb6gaUjabb+gaVjabbsha0jabbggaHjabbsha0jabbwgaLjabbohaZnaaCaaaleqabaaccaGae8NeI0ccbaGae4xmaedaaaqaamaaoGcameqabaaaliaawkziaaqabaaaaa@3CDD@	StringlikelyProt2syn→ MathType@MTEF@5@5@+=feaafiart1ev1aaatCvAUfKttLearuWrP9MDH5MBPbIqV92AaeXatLxBI9gBaebbnrfifHhDYfgasaacH8akY=wiFfYdH8Gipec8Eeeu0xXdbba9frFj0=OqFfea0dXdd9vqai=hGuQ8kuc9pgc9s8qqaq=dirpe0xb9q8qiLsFr0=vr0=vr0dc8meaabaqaciaacaGaaeqabaqabeGadaaakeaadaWfqaqaaiabbYgaSjabbMgaPjabbUgaRjabbwgaLjabbYgaSjabbMha5jabbcfaqjabbkhaYjabb+gaVjabbsha0jabbkdaYiabbohaZjabbMha5jabb6gaUbWcbaWaa4akaWqabeaaaSGaayPKHaaabeaaaaa@415B@	StringpossibleProt2syn→ MathType@MTEF@5@5@+=feaafiart1ev1aaatCvAUfKttLearuWrP9MDH5MBPbIqV92AaeXatLxBI9gBaebbnrfifHhDYfgasaacH8akY=wiFfYdH8Gipec8Eeeu0xXdbba9frFj0=OqFfea0dXdd9vqai=hGuQ8kuc9pgc9s8qqaq=dirpe0xb9q8qiLsFr0=vr0=vr0dc8meaabaqaciaacaGaaeqabaqabeGadaaakeaadaWfqaqaaiabbchaWjabb+gaVjabbohaZjabbohaZjabbMgaPjabbkgaIjabbYgaSjabbwgaLjabbcfaqjabbkhaYjabb+gaVjabbsha0jabbkdaYiabbohaZjabbMha5jabb6gaUbWcbaWaa4akaWqabeaaaSGaayPKHaaabeaaaaa@4417@
GeneSynhasTerm→ MathType@MTEF@5@5@+=feaafiart1ev1aaatCvAUfKttLearuWrP9MDH5MBPbIqV92AaeXatLxBI9gBaebbnrfifHhDYfgasaacH8akY=wiFfYdH8Gipec8Eeeu0xXdbba9frFj0=OqFfea0dXdd9vqai=hGuQ8kuc9pgc9s8qqaq=dirpe0xb9q8qiLsFr0=vr0=vr0dc8meaabaqaciaacaGaaeqabaqabeGadaaakeaadaWfqaqaaiabbIgaOjabbggaHjabbohaZjabbsfaujabbwgaLjabbkhaYjabb2gaTbWcbaWaa4akaWqabeaaaSGaayPKHaaabeaaaaa@37DC@	TrainFilehasTerm→ MathType@MTEF@5@5@+=feaafiart1ev1aaatCvAUfKttLearuWrP9MDH5MBPbIqV92AaeXatLxBI9gBaebbnrfifHhDYfgasaacH8akY=wiFfYdH8Gipec8Eeeu0xXdbba9frFj0=OqFfea0dXdd9vqai=hGuQ8kuc9pgc9s8qqaq=dirpe0xb9q8qiLsFr0=vr0=vr0dc8meaabaqaciaacaGaaeqabaqabeGadaaakeaadaWfqaqaaiabbIgaOjabbggaHjabbohaZjabbsfaujabbwgaLjabbkhaYjabb2gaTbWcbaWaa4akaWqabeaaaSGaayPKHaaabeaaaaa@37DC@	GeneIdsynonym→ MathType@MTEF@5@5@+=feaafiart1ev1aaatCvAUfKttLearuWrP9MDH5MBPbIqV92AaeXatLxBI9gBaebbnrfifHhDYfgasaacH8akY=wiFfYdH8Gipec8Eeeu0xXdbba9frFj0=OqFfea0dXdd9vqai=hGuQ8kuc9pgc9s8qqaq=dirpe0xb9q8qiLsFr0=vr0=vr0dc8meaabaqaciaacaGaaeqabaqabeGadaaakeaadaWfqaqaaiabbohaZjabbMha5jabb6gaUjabb+gaVjabb6gaUjabbMha5jabb2gaTbWcbaWaa4akaWqabeaaaSGaayPKHaaabeaaaaa@386E@
StringhasSpan−1→ MathType@MTEF@5@5@+=feaafiart1ev1aaatCvAUfKttLearuWrP9MDH5MBPbIqV92AaeXatLxBI9gBaebbnrfifHhDYfgasaacH8akY=wiFfYdH8Gipec8Eeeu0xXdbba9frFj0=OqFfea0dXdd9vqai=hGuQ8kuc9pgc9s8qqaq=dirpe0xb9q8qiLsFr0=vr0=vr0dc8meaabaqaciaacaGaaeqabaqabeGadaaakeaadaWfqaqaaiabbIgaOjabbggaHjabbohaZjabbofatjabbchaWjabbggaHjabb6gaUnaaCaaaleqabaaccaGae8NeI0ccbaGae4xmaedaaaqaamaaoGcameqabaaaliaawkziaaqabaaaaa@39D1@	LabelshasSpanType→ MathType@MTEF@5@5@+=feaafiart1ev1aaatCvAUfKttLearuWrP9MDH5MBPbIqV92AaeXatLxBI9gBaebbnrfifHhDYfgasaacH8akY=wiFfYdH8Gipec8Eeeu0xXdbba9frFj0=OqFfea0dXdd9vqai=hGuQ8kuc9pgc9s8qqaq=dirpe0xb9q8qiLsFr0=vr0=vr0dc8meaabaqaciaacaGaaeqabaqabeGadaaakeaadaWfqaqaaiabbIgaOjabbggaHjabbohaZjabbofatjabbchaWjabbggaHjabb6gaUjabbsfaujabbMha5jabbchaWjabbwgaLbWcbaWaa4akaWqabeaaaSGaayPKHaaabeaaaaa@3D30@	EntityTypehasSpanType−1→ MathType@MTEF@5@5@+=feaafiart1ev1aaatCvAUfKttLearuWrP9MDH5MBPbIqV92AaeXatLxBI9gBaebbnrfifHhDYfgasaacH8akY=wiFfYdH8Gipec8Eeeu0xXdbba9frFj0=OqFfea0dXdd9vqai=hGuQ8kuc9pgc9s8qqaq=dirpe0xb9q8qiLsFr0=vr0=vr0dc8meaabaqaciaacaGaaeqabaqabeGadaaakeaadaWfqaqaaiabbIgaOjabbggaHjabbohaZjabbofatjabbchaWjabbggaHjabb6gaUjabbsfaujabbMha5jabbchaWjabbwgaLnaaCaaaleqabaaccaGae8NeI0ccbaGae4xmaedaaaqaamaaoGcameqabaaaliaawkziaaqabaaaaa@3F31@

The final set of features used by learning system, in the format "t→ℓ'
 MathType@MTEF@5@5@+=feaafiart1ev1aaatCvAUfKttLearuWrP9MDH5MBPbIqV92AaeXatLxBI9gBaebbnrfifHhDYfgasaacH8akY=wiFfYdH8Gipec8Eeeu0xXdbba9frFj0=OqFfea0dXdd9vqai=hGuQ8kuc9pgc9s8qqaq=dirpe0xb9q8qiLsFr0=vr0=vr0dc8meaabaqaciaacaGaaeqabaqabeGadaaakeaaieGacqWFIaGicqWG0baDdaGdKaWcbaGaeS4eHWgabeGccaGLsgcacqGGNaWjaaa@3290@, where *t *is a node type, and ℓ is an edge label. The edge labels *likelyProtNER *and *possibleProtNER *are shortcut representing the baseline NER methods. The edge labels *likelyProt2syn *and *possibleProt2syn *are links between extracted protein names and softTFIDF-similar protein synonyms.

We then recursively define another vector function **F **which aggregates these feature primitive functions over a walk that starts at node *x *and walks to node *z *in exactly *d *steps, as follows:

F(x→=0z)=0F(x→=dz)=∑y(∑ℓPr⁡(ℓ|x)⋅Pr⁡(y|x,ℓ)⋅f(y→ℓz))⋅Q(y→=d−1z)
 MathType@MTEF@5@5@+=feaafiart1ev1aaatCvAUfKttLearuWrP9MDH5MBPbIqV92AaeXatLxBI9gBaebbnrfifHhDYfgasaacH8akY=wiFfYdH8Gipec8Eeeu0xXdbba9frFj0=OqFfea0dXdd9vqai=hGuQ8kuc9pgc9s8qqaq=dirpe0xb9q8qiLsFr0=vr0=vr0dc8meaabaqaciaacaGaaeqabaqabeGadaaakeaafaqaaeGabaaabaacbeGae8NrayKaeiikaGIaemiEaG3aa4ajaSqaaiabg2da9iabicdaWaqabOGaayPKHaGaemOEaONaeiykaKIaeyypa0Jae8hmaadabaGae8NrayKaeiikaGIaemiEaG3aa4ajaSqaaiabg2da9iabdsgaKbqabOGaayPKHaGaemOEaONaeiykaKIaeyypa0ZaaabeaeaacqGGOaakdaaeqaqaaiGbccfaqjabckhaYjabcIcaOiabloriSjabcYha8jabdIha4jabcMcaPiabgwSixlGbccfaqjabckhaYjabcIcaOiabdMha5jabcYha8jabdIha4jabcYcaSiabloriSjabcMcaPiabgwSixlab=zgaMjabcIcaOiabdMha5naaoqcaleaacqWItecBaeqakiaawkziaiabdQha6jabcMcaPiabcMcaPiabgwSixlabdgfarjabcIcaOiabdMha5naaoqcaleaacqGH9aqpcqWGKbazcqGHsislcqaIXaqmaeqakiaawkziaiabdQha6jabcMcaPaWcbaGaeS4eHWgabeqdcqGHris5aaWcbaGaemyEaKhabeqdcqGHris5aaaaaaa@7946@

Finally, we can define

F(z|x)=γ∑d=1dmax(1−γ)dF(x→=dz)
 MathType@MTEF@5@5@+=feaafiart1ev1aaatCvAUfKttLearuWrP9MDH5MBPbIqV92AaeXatLxBI9gBaebbnrfifHhDYfgasaacH8akY=wiFfYdH8Gipec8Eeeu0xXdbba9frFj0=OqFfea0dXdd9vqai=hGuQ8kuc9pgc9s8qqaq=dirpe0xb9q8qiLsFr0=vr0=vr0dc8meaabaqaciaacaGaaeqabaqabeGadaaakeaaieqacqWFgbGrcqGGOaakcqWG6bGEcqGG8baFcqWG4baEcqGGPaqkcqGH9aqpiiGacqGFZoWzdaaeWbqaaiabcIcaOiabigdaXiabgkHiTiab+n7aNjabcMcaPmaaCaaaleqabaGaemizaqgaaaqaaiabdsgaKjabg2da9iabigdaXaqaaiabdsgaKnaaBaaameaaieGacqqFTbqBcqqFHbqycqqF4baEaeqaaaqdcqGHris5aOGae8NrayKaeiikaGIaemiEaG3aa4ajaSqaaiabg2da9iabdsgaKbqabOGaayPKHaGaemOEaONaeiykaKcaaa@521E@

The algorithm of Table 3 was modified to compute (an approximation to) **F**(*z*|*x*) in the process of approximating *Q*(*z*|*x*). In our implementation, this approximately doubles the cost of the computation. We modify this approximated feature vector **F **in two ways. First, we add a final feature function which records the final score *Q*(*z*|*x*) for *z*. Second, we convert all feature values to their logarithms.

In our learning experiments, we iterated over all of the examples 200 times.

## Availability and requirements

The source code for the software discussed in this paper is available as an additional file with the manuscript (see Additional File 1). The code is written in Java, and has been tested on Windows and Linux. The code requires installation of Minorthird, an open-source package which is available from mi-northird.sourceforge.net.

## Authors' contributions

EM implemented some of the graph-search and learning framework, contributed to the recall-precision adjustment of the NER methods. WC performed the remainder of the implementation and experimentation. All authors read and approved the final manuscript.

## Supplementary Material

Additional File 1 The code used for graph-search and learning-to-rank in this paper has been submitted as an additional file: a gzip-compressed tar file, with all code under an open source license. The code requires installation of Minorthird, another open-source package. The README.txt file in the top-level directory details how to compile and use the code. The code for combining NER and soft matching is implemented a special case of the graph search that uses a restricted set of paths through the graph, realized using the class SoftDictEntitySearcher. *Code availability and requirements *All code is implemented in Java, and has been verified to run on both Windows XP (with cygwin) and Red Hat Linux environments. The most recent version of the code is available via anonymous CVS from the authors, using the following commands:
export CVS_RSH=ssh
export cvsroot=:pserver:anonymous@raff.ml.cmu.edu:/usr1/cvsroot
% cvs login
(anything as a password)
% cvs checkout ghirl Click here for file
